# Supplementary material for: The whack-a-mole governance challenge for AI-enabled synthetic biology: literature review and emerging frameworks
Source: Front Bioeng Biotechnol. 2024 Feb 28;12:1359768. doi: 10.3389/fbioe.2024.1359768 (PMC10933118; doi:10.3389/fbioe.2024.1359768)
Supplement: Supplementary file 2 [file Table2.DOCX]

|  | **Author(s)** | **Title** | **Type** | **Sector(s)** | **Keywords** |
| --- | --- | --- | --- | --- | --- |
| 1 | [(Hagendorff, 2021)](https://paperpile.com/c/L9hHvg/e7j0) | Forbidden knowledge in machine learning reflections on the limits of research and publication | Peer reviewed article |  | Forbidden knowledge,  Machine learning,  Artificial intelligence,  Governance,  Dual-use,  Publication norms, open access, precautionary principle, AI ethics |
| 2 | [(Linkov, Trump, Anklam, *et al.*, 2018)](https://paperpile.com/c/L9hHvg/ohqD) | Comparative, collaborative, and integrative risk governance for emerging technologies | Peer reviewed article |  | Synthetic biology,  Biotechnology,  Nanotechnology,  Governance,  Risk assessment,  Policy,  Decision analysis,  Regulations, key enabling technologies, emerging technologies, risk governance, |
| 3 | [(Blok and von Schomberg, 2023)](https://paperpile.com/c/L9hHvg/lewG) | Putting Responsible Research and Innovation into Practice | Book | EU | Responsible Research and Innovation (RRI), Research and Innovation (R & I), disruptive technologies, synthetic biology, artificial intelligence, robotics, science, pandemic, responsible AI, responsible agroecology, Horizon 2020, science and society, civil society, science policy, EU, EU Framework Programme for Research and Innovation |
| 4 | [(Reynolds, 2021)](https://paperpile.com/c/L9hHvg/zi39) | Engineering biological diversity: the international governance of synthetic biology, gene drives, and de-extinction for conservation | Peer reviewed article |  | Biological diversity, governance, synbio governance, gene drives, conservation, international law, national law, self-governance, conservationist synthetic biology, environmental risks, Convention on Biological Diversity (CBD), emerging technologies, |
| 5 | [(Xiao *et al.*, 2023)](https://paperpile.com/c/L9hHvg/WxyO) | Generative Artificial Intelligence GPT-4 Accelerates Knowledge Mining and Machine Learning for Synthetic Biology | Peer reviewed article |  | Yarrowia lipolytica; feature selection; human intervention; natural language processing; prompt engineering; transfer learning, information extraction, biomanufacturing, random forest model, machine learning (ML), generative AI, metabolic engineering, synthetic biology |
| 6 | [(Bray, 2023)](https://paperpile.com/c/L9hHvg/Sihx) | Artificial Intelligence and Synthetic Biology Are Not Harbingers of Doom | Think tank |  | AI, synthetic biology, benevolence, pathogens, bioterrorism, precursor behaviors, LLMs |
| 7 | [(Carter *et al.*, 2023)](https://paperpile.com/c/L9hHvg/yzyO) | The Convergence of Artificial Intelligence and the Life Sciences | Think tank |  | Convergence, AI, synthetic biology, bioscience, bioengineering, AI-bio capabilities, malicious actors, biological catastrophe, biological agents, pathogens, toxins, policy recommendations, AI guardrails, biosecurity, governance |
| 8 | [(Tong and Zhang, 2023)](https://paperpile.com/c/L9hHvg/Q17l) | Discovering the next decade's synthetic biology research trends with ChatGPT | P |  | chatGPT, synthetic biology research trends, conversation with chatGPT |
| 9 | [(Grinbaum and Adomaitis, 2023)](https://paperpile.com/c/L9hHvg/A148) | Dual Use Concerns of Generative AI and Large Language Models | preprint |  | DURC, LLMs, Dual Use Research of Concern (DURC) framework, AI governance, gain-of-function (GOF), public safety, National Security  Advisory Board for Biosecurity (NSABB), Fink report, shared responsibility |
| 10 | [(Yamagata, 2023)](https://paperpile.com/c/L9hHvg/duUO) | SynBio: A Journal for Advancing Solutions to Global Challenges | Editorial |  | Synbio, global challenges, synbio applications, AI |
| 11 | [(Achim and Zhang, 2022)](https://paperpile.com/c/L9hHvg/nY2v) | Exploring the social, ethical, legal, and responsibility dimensions of artificial intelligence for health-a new column in Intelligent Medicine | Peer |  | Intelligent medicine, AI in medicine, AI in healthcare, AI for health, AI governance, AI systems, ELSI/A, responsible research and innovation (RRI), open science |
| 12 | [(Li *et al.*, 2023)](https://paperpile.com/c/L9hHvg/jsU6) | ChatGPT in Healthcare: A Taxonomy and Systematic Review |  | preprint | chatGPT, taxonomy, systematic review, natural language processing (NLP), OpenAI, AI, PubMed, biomedical datasets, medical applications, research gaps, clinical workflow, medical research, medical education, consultation, triage, translation, multimodal |
| 13 | [(Dixon, Walker and Pretorius, 2023)](https://paperpile.com/c/L9hHvg/BaC7) | Visioning synthetic futures for yeast research within the context of current global techno-political trends |  |  | metagenome; minimal genome; pan-genome; supernumerary neochromosome; synthetic communities; synthetic genome, yeast research, techno-political trends, Saccharomyces cerevisiae, AI-enabled tools for the life sciences, applied science, basic science, convergence, synthetic yeast research |
| 14 | [(Mahesh *et al.*, 2023)](https://paperpile.com/c/L9hHvg/j5SG) | Viable remediation techniques to cleansing wastewaters comprising endocrine-disrupting compounds |  | peer | Endocrine-disrupting chemicals (EDCs), AI system, synthetic biology, biodegradation, wastewater |
| 15 | [(Xu *et al.*, 2022)](https://paperpile.com/c/L9hHvg/3i6a) | Bibliometric analysis of artificial intelligence for biotechnology and applied microbiology: Exploring research hotspots and frontiers | United States, Chinese Academy of Science | peer | applied microbiology; artificial intelligence; bibliometric; biotechnology; deep learning; machine learning, disease diagnostics, drug research and development, functional genomics, biomarker recognition, medical imaging diagnostics, Web of Science Core Collection (WoSCC), microRNA, protein-protein interactions (PPIs) |
| 16 | [(Wu *et al.*, 2022)](https://paperpile.com/c/L9hHvg/8DaR) | Research Trends in the Application of Artificial Intelligence in Oncology: A Bibliometric and Network Visualization Study |  | China | artificial intelligence; bibliometrics; cancer; deep learning; hotspots, AI in cancer, AI in oncology, AI in radiomics, AI in precision oncology, precision medicine, Science Citation Index Expanded, Scientific Reports, PloS, tissue microarray, tissue segmentation, artificial neural network |
| 17 | [(Shi *et al.*, 2022)](https://paperpile.com/c/L9hHvg/x62f) | Data-Driven Synthetic Cell Factories Development for Industrial Biomanufacturing |  | peer | artificial intelligence (AI), machine learning (ML), artificial cell factories, industrial biomanufacturing, biomanufacturing, cell factories, protein function, non-natural biosynthesis pathways, pathway optimization, synthetic strains |
| 18 | [(Helmy, Smith and Selvarajoo, 2020)](https://paperpile.com/c/L9hHvg/jL1H) | Systems biology approaches integrated with artificial intelligence for optimized metabolic engineering |  | peer | Artificial intelligence; Food industry; Machine learning; Metabolic engineering; Systems biology, metabolic pathways, compound libraries, multi-omics, transcriptomics, proteomics, metabolomics, holistic understanding, industrial biotechnology, food-related substances, microorganisms |
| 19 | [(Hoffmann, 2023)](https://paperpile.com/c/L9hHvg/iCj0) | Designer genes courtesy of artificial intelligence |  | peer | Drosophila; RNA polymerase II; core promoter; gene expression; transcription, machine learning, statistical learning model |
| 20 | [(Capponi and Daniels, 2023)](https://paperpile.com/c/L9hHvg/9A8g) | Harnessing the power of artificial intelligence to advance cell therapy |  | peer | cell signaling; cell therapy; machine learning; signaling motifs; synthetic biology, Artificial intelligence (AI), machine learning (ML), predictive models, experimental approaches, genome annotation, protein structure prediction, modular cell therapy technologies |
| 21 | [(Y. Zeng *et al.*, 2022)](https://paperpile.com/c/L9hHvg/JbY7) | Advanced genome-editing technologies enable rapid and large-scale generation of genetic variants for strain engineering and synthetic biology |  | peer | Genome-editing, genetic variants, strain engineering, synthetic biology, CRISPR, RNA-based tools, AI-optimization algorithms, biomanufacturing, biosensing, high-throughput phenotyping, biological engineering |
| 22 | [(Mervin *et al.*, 2021)](https://paperpile.com/c/L9hHvg/BCrh) | Uncertainty quantification in drug design |  | peer | Uncertainty quantification, drug design, AI, drug-design, algorithms, autonomous decision-making, chemistry automation, decision-making, molecular de novo generation, synthetic route prediction, property predictions |
| 23 | [(Yu, Wang and Zheng, 2022)](https://paperpile.com/c/L9hHvg/wrAc) | Uncertainty quantification: Can we trust artificial intelligence in drug discovery? |  | peer | Applied computing; Artificial intelligence; Drugs, Uncertainty quantification, drug design, human trust, applicability domain, training set, autonomous drug designing |
| 24 | [(Koskinen, 2020)](https://paperpile.com/c/L9hHvg/5QHt) | Could We Really Be Made of Swiss Cheese? Xenobiology as an Engineering Epistemology for Biological Realization |  | peer | bioorganic chemistry; multiple realizability; philosophy of science; synthetic biology; xenobiology, digital minds |
| 25 | [(Martin *et al.*, 2023)](https://paperpile.com/c/L9hHvg/sEli) | Perspectives for self-driving labs in synthetic biology |  | peer | self-driving labs (SDLs), synthetic biology, |
| 26 | [(Lawrence, 2019)](https://paperpile.com/c/L9hHvg/DVLE) | Advanced bioscience and AI: debugging the future of life |  | peer | AI, homo sapiens, sapient beings, sapient AIs, artificial intelligence; bioethics; new life; personhood; synthetic biology |
| 27 | [(Ebrahimkhani and Levin, 2021)](https://paperpile.com/c/L9hHvg/i3wh) | Synthetic living machines: A new window on life |  | peer | bioengineering; developmental biology; synthetic biology, synthetic living machines, molecular genetics, morphogenesis, synthetic morphogenesis, meso-scale morpogenesis, self-assembly, emergent self-organization, synthetic anatomies, evolutionary cell biology |
| 28 | [(Bongard and Levin, 2023)](https://paperpile.com/c/L9hHvg/uLoS) | There's Plenty of Room Right Here: Biological Systems as Evolved, Overloaded, Multi-Scale Machines |  | peer | artificial intelligence; artificial life; biology; computer science; evolution; machine learning; robot, biomimetics, biological systems, multi-scale machines, intelligent machines, physical computing, polycomputing, overloading, regenerative medicine |
| 29 | [(Blackiston *et al.*, 2023)](https://paperpile.com/c/L9hHvg/AON5) | Biological Robots: Perspectives on an Emerging Interdisciplinary Field |  | peer | animal cap; biorobot; computer science; developmental biology; embryo; robot; synthetic bioengineering; xenobot, regenerative medicine, synthetic living machines |
| 30 | [(Webster-Wood *et al.*, 2022)](https://paperpile.com/c/L9hHvg/uVfe) | Biohybrid robots: recent progress, challenges, and perspectives |  | peer | biohybrid; cyborg; living machines, biohybrid robotics, microorganism bots, sperm-bots, tissue-based robots, autonomous living machines |
| 31 | [(Li *et al.*, 2022)](https://paperpile.com/c/L9hHvg/OMXN) | An Account of Models of Molecular Circuits for Associative Learning with Reinforcement Effect and Forced Dissociation |  | peer | associative learning, molecular circuits, synthetic biology, mathematical modelling, Hill equation, Pavlov’s dog, reinforcement, dissociation, nondimensionalisation |
| 32 | [(Nesbeth *et al.*, 2016)](https://paperpile.com/c/L9hHvg/w4LE) | Synthetic biology routes to bio-artificial intelligence |  | peer | artificial intelligence, gene networks, synthetic biological circuits, synthetic biology, bio-artificial intelligence (BI) |
| 33 | [(Kumar, Sinha and Shukla, 2022)](https://paperpile.com/c/L9hHvg/NKUg) | Artificial intelligence and synthetic biology approaches for human gut microbiome |  | peer | Artificial intelligence; CRISPR-Cas; gut microbiome; nutraceutical; probiotics; synthetic biology, assisted synthetic biology, Clustered Regularly Interspaced Short Palindromic Repeats (CRISPR-Cas) |
| 34 | [(Wong, de la Fuente-Nunez and Collins, 2023)](https://paperpile.com/c/L9hHvg/fxFc) | Leveraging artificial intelligence in the fight against infectious diseases |  | peer | AI, infectious disease, pathogen, synthetic biology, anti-infective drug discovery. Reverse vaccinology, epidemiology, explainable ML, infection biology, pandemics, antimicrobial resistance |
| 35 | [(Zhang *et al.*, 2023)](https://paperpile.com/c/L9hHvg/Gffq) | Enabling technology and core theory of synthetic biology |  | peer | synthetic biology,  quantitative synthetic biology,  genome synthesis and assembly,  DNA storage,  molecular evolution,  de novo design,  computer-aided design,  cell engineering,  gene circuit,  chassis cell,  artificial intelligent (AI),  Biofoundry, cell-free synthetic biology |
| 36 | [(Nelson *et al.*, 2021)](https://paperpile.com/c/L9hHvg/vA0S) | Foresight in Synthetic Biology and Biotechnology Threats |  | Book chapter | Foresight, biothreats, synthetic biology, risk landscape, risk mitigation, NATO |
| 37 | [(Endy, 2005)](https://paperpile.com/c/L9hHvg/Ul6q) | Foundations for engineering biology |  | peer | Synthetic biology, engineered biological systems, foundational technologies, open science, standardization, biorisks, biological risks |
| 38 | [(Alexander Hamilton *et al.*, 2021)](https://paperpile.com/c/L9hHvg/a9k6) | Opportunities, Challenges, and Future Considerations for Top-Down Governance for Biosecurity and Synthetic Biology |  |  | Biosecurity, synthetic biology, top-down governance, laissez-faire governance, stewardship governance, bioeconomy |
| 39 | [(Kahl and Endy, 2013)](https://paperpile.com/c/L9hHvg/HMs1) | A survey of enabling technologies in synthetic biology |  |  | Synthetic biology, Biological engineering, Enabling technologies, Survey, Intellectual property rights, Licensing, Regulation |
| **40** | [(Holzinger *et al.*, 2023)](https://paperpile.com/c/L9hHvg/SMy4) | AI for life: Trends in artificial intelligence for biotechnology |  | Editorial | Artificial Intelligence, Biotechnology, Deep Learning, Digital Transformation, Machine Learning, drug discovery, drug safety, functional and structural genomics, proteomics, metabolomics, pharmacology, pharmacogenetics, and pharmacogenomics, agricultural biotechnology, AI in forest biotechnology, AI in medical biotechnology, AI in Animal biotechnology, AI in bioinformatics, trustworthy AI, AI methods, Human-AI interfaces, AI ethics, AI fairness, AI trust, personalized medicine, disease prediction, biomedical image analysis, Digital Information Processing System, AI ecosystems |
| 44 | [(O’Brien and Nelson, 2020)](https://paperpile.com/c/L9hHvg/aNfE) | Assessing the Risks Posed by the Convergence of Artificial Intelligence and Biotechnology |  |  | Risk assessment, Artificial intelligence, Biotechnology, Biosecurity, NASEM Framework, Tucker Framework, synthetic biology, vulnerabilities, deep learning, precision maladies, pathogens, engineered pathogens, AI systems, AAAS-FBI-UNICRI Framework, Tucker-Koblentz framework, biosecurity |
| 41 | [(A Dixon, C Curach and Pretorius, 2020)](https://paperpile.com/c/L9hHvg/D1u4) | Bio-informational futures  The convergence of artificial intelligence and synthetic biology |  |  | Biofoundries, AI, synthetic biology, technology platforms, policy problems, bio-informational future, future, genome foundries, modular laboratory equipment, MIT Foundry, Gingko Bioworks, Amyris, the London Biofoundry, biological devices, productivity, Global Biofoundries Alliance (GBA) |
| **42** | [(Hillson *et al.*, 2019)](https://paperpile.com/c/L9hHvg/KAAy) | Building a global alliance of biofoundries |  |  | Metabolic engineering, Synthetic biology, AI, automation, workflows, tools, Design-Build-Test-Learn (DTBL), machine learning, testing, large-scale genetic designs, biomedical applications, biodesign rules, reprogramming living cells, complexity of living systems, product prototyping, commercial process validation, biomanufacturing, process engineering, high-throughput equipment |
| 43 | [(Oliveira and Densmore, 2022)](https://paperpile.com/c/L9hHvg/4HYA) | Hardware, Software, and Wetware Codesign Environment for Synthetic Biology |  |  | Synthetic biology, wetware codesign, biodesign automation, genetic circuits, engineered genetic circuits, AI, software, engineering framework, synthetic biological systems, DNA biofoundries, cloud laboratories, standards |
| 44 | [(Kitano *et al.*, 2023)](https://paperpile.com/c/L9hHvg/9um6) | Synthetic biology: Learning the way toward high-precision biological design |  |  | Synthetic biology, machine learning, genetics, human genomics, synthetic genomics, bioengineering, biologists, DNA synthesis, biomanufacturing, design-build-test-learn (DBTL) cycle |
| 45 | [(Damiano and Stano, 2023)](https://paperpile.com/c/L9hHvg/JME3) | Explorative Synthetic Biology in AI: Criteria of Relevance and a Taxonomy for Synthetic Models of Living and Cognitive Process |  |  | Artificial intelligence, synthetic biology, epistemology of synthetic models, taxonomy of synthetic models, synthetic method, criteria of relevance |
| **46** | [(Eslami *et al.*, 2022)](https://paperpile.com/c/L9hHvg/HxPt) | Artificial intelligence for synthetic biology |  | peer | AI, applied computing, computational biology, computing methodologies, life and medical sciences, metabolic engineering, experiment automation, multimodality, design on living cells |
| 47 | [(Ataii *et al.*, 2023)](https://paperpile.com/c/L9hHvg/jADd) | Enabling AI in Synthetic Biology through Construction File Specification |  |  | Construction File (CF) specification, Python, ChatGPT, GPT-4, modularization, automation, collaboration, synthetic biology, AI, AI-assisted workflows, human-driven design |
| 48 | [(DiEuliis *et al.*, 2019)](https://paperpile.com/c/L9hHvg/uxEg) | Does Biotechnology Pose New Catastrophic Risks? |  | Chapter | Biotechnology, catastrophic risks, synthetic biology, biosecurity, genetic engineering, dual-use capabilities, tools, preparedness, emerging technologies, viral genomes, recreating pathogens, resurrecting extinct species, infectious disease, biosurveillance, bioagents, bioweapons |
| 49 | [(Mampuys and Brom, 2018)](https://paperpile.com/c/L9hHvg/qBt2) | Emerging crossover technologies: How to organize a biotechnology that becomes mainstream? |  |  | Biotechnology, Emerging technologies,  Synthetic biology,  Regulations,  Europe |
| **50** | [(Murashov, Howard and Schulte, 2020)](https://paperpile.com/c/L9hHvg/yQG7) | Synthetic Biology Industry: Biosafety Risks to Workers |  | Chapter | Biosafety risks, workers, synthetic biology industry, manufacturing, biomanufacturing, biotechnology sector, USA, US economy, bioethics, risk mitigation, health surveillance, proactive risk management, prevention-through-design principles, dynamic guidance, adverse health effects, NIOSH, emerging technologies |
| **51** | [(Pio-Lopez, 2021)](https://paperpile.com/c/L9hHvg/UR9c) | The rise of the biocyborg: synthetic biology, artificial chimerism and human enhancement |  |  | Biopolitics, biocyborg, synthetic biology, human enhancement, gene editing, self-experimentation, biohacking, cyborg, chimerism, bioeconomy, human being, human species, hybridity, social practices, implantables, active medical devices, non-Darwinian human evolution |
| **52** | [(Hanson and Lorenzo, 2023)](https://paperpile.com/c/L9hHvg/taYX) | Synthetic Biology─High Time to Deliver? |  | peer | Bacteria, Bioengineering and biotechnology, Biotechnology, Circuits, Genetics, critique, biologization, practical solutions, real-world problems, synbio hype, tools, bioeconomy, industrialization , overspecialization, optimization, biofoundry |
| 53 | [(Wang and Zhang, 2019)](https://paperpile.com/c/L9hHvg/3ylQ) | Synthetic biology: Recent progress, biosafety and biosecurity concerns, and possible solutions |  |  | Synthetic biology, Biosafety, Biosecurity, Ethics, Genetic firewall, Genetic safeguard, Code of conduct, Governance regulation, iGEM, international genetically engineered machine competition, Biological and Toxin Weapons Convention (BWC) |
| 54 | [(Li *et al.*, 2021)](https://paperpile.com/c/L9hHvg/Gq8K) | Advances in Synthetic Biology and Biosafety Governance |  |  | synthetic biology, artificial life, biosafety, regulation and legislation, public health emergency response system, pandemic control strategies. Governance, biological risks, biorisks, food security, ecological sustainability, industrialization, pathogens, biosafety governance, biosecurity, USA, FDCA, CDC, NIH, DURC, pandemics, control |
| 55 | [(Trump *et al.*, 2022)](https://paperpile.com/c/L9hHvg/vWiK) | Governing biotechnology to provide safety and security and address ethical, legal, and social implications |  |  | biotechnology, ELSI, policy, governance, safety-by-design, synthetic biology, ethical, legal, and social implications, risk, AI, technology modernization, technology advancement |
| **56** | [(Sundaram, Ajioka and Molloy, 2023)](https://paperpile.com/c/L9hHvg/yNII) | Synthetic biology regulation in Europe: containment, release and beyond |  |  | regulation, EU, synthetic biology, biotechnology, containment, genetic engineering, regulatory frameworks, bioengineering, GM, GMO regulation, biosensor, synbio products, risk assessments |
| **57** | [(Trump *et al.*, 2019)](https://paperpile.com/c/L9hHvg/0zpn) | Co-evolution of physical and social sciences in synthetic biology |  | peer | Synthetic biology, risk, governance, review, policy |
| 58 | [(Vallejos-Romero *et al.*, 2021)](https://paperpile.com/c/L9hHvg/7J7d) | Social sciences, risks and synthetic biology: A review of the literature |  | peer | synthetic biology, risks, regulation, governance, controversies |
| 59 | [(Sarkar *et al.*, 2023)](https://paperpile.com/c/L9hHvg/yoYo) | Artificial Intelligence and Machine Learning Technology Driven Modern Drug Discovery and Development |  |  | artificial intelligence; machine learning; drug discovery; virtual screening; QSAR; QSPR; algorithms; neural networks |
| 60 | [(Jafari *et al.*, 2023)](https://paperpile.com/c/L9hHvg/CQEO) | Merging machine learning and bioelectronics for closed-loop control of biological systems and homeostasis |  |  | Machine learning, bioelectronics, artificial technology, biological systems, bioelectrics, sensor data, actuator data, biological system responses |
| 61 | [(Vaseashta, 2023)](https://paperpile.com/c/L9hHvg/Ypyt) | Existential Risks Associated with Dual-Use Technologies |  |  | dual-use technologies, catastrophic risk, existential risk, threat intelligence, nanobiotechnology |
| 62 | [(Stano, 2023)](https://paperpile.com/c/L9hHvg/jeJ2) | Chemical Systems for Wetware Artificial Life: Selected Perspectives in Synthetic Cell Research |  |  | artificial cells; artificial intelligence; artificial life; autopoiesis; chemical AI; cognition; synthetic biology; synthetic cells; semantic information; systems chemistry |
| 63 | [(Qiu and Wei, 2023)](https://paperpile.com/c/L9hHvg/XDam) | Artificial intelligence-aided protein engineering: from topological data analysis to deep protein language models |  |  | topological data analysis, protein language models, protein engineering, deep learning and machine learning, ML, NLP, AI |
| 64 | [(‘Generating “smarter” biotechnology’, 2023)](https://paperpile.com/c/L9hHvg/zNOH) | Generating ‘smarter’ biotechnology |  |  | Generative AI, biotechnology, |
| 65 | [(Getz and Dellaire, 2018)](https://paperpile.com/c/L9hHvg/Uulp) | Angels and Devils: Dilemmas in Dual-Use Biotechnology |  |  | dual use, dual-use policy  CRISPR/Cas9,  research of concern,  ethics education, synthetic virology, gene-drive technology, human gene editing |
| 66 | [(J. B. Sandbrink, 2023)](https://paperpile.com/c/L9hHvg/S8fi) | Artificial intelligence and biological misuse: Differentiating risks of language models and biological design tools |  |  | DURC, LLMs, GPT-4, AI, biological misuse, risk, biorisk, biological design tools (BDTs), |
| 67 | [(Soice *et al.*, 2023)](https://paperpile.com/c/L9hHvg/TGC8) | Can large language models democratize access to dual-use biotechnology? |  |  | LLMs, dual-use, democratized access, reverse genetics, DNA synthesis, LLM chatbots, robotic cloud laboratories, MIT, pandemic-class agents |
| 68 | [(D’Alessandro, Lloyd and Sharadin, 2023)](https://paperpile.com/c/L9hHvg/Sz0r) | Large Language Models and Biorisk |  | Commentary | Biorisk, LLMs, ML, AI, ML model misuse, dangerous bioagents, lab security measures, biomedical ML systems, bad actors, biomedical AI assistants, toxic compounds, mass misinformation campaigns, prompt engineering, online misinformation, machine-generated examples, red-teaming, sandboxed environments, BSL requirements, biosecurity safety level (BSL), information security |
| 69 | [(Urbina *et al.*, 2022)](https://paperpile.com/c/L9hHvg/shnX) | Dual use of artificial-intelligence-powered drug discovery |  | Comment | Dual use, AI, drug discovery, toxic molecules |
| 70 | [(Mökander *et al.*, 2022)](https://paperpile.com/c/L9hHvg/Z9FJ) | Challenges and best practices in corporate AI governance: Lessons from the biopharmaceutical industry |  |  | artificial intelligence; AstraZeneca, case study; ethics; governance; implementation; lessons learned; practice |
| 71 | [(de Pedraza and Vollbracht, 2023)](https://paperpile.com/c/L9hHvg/oLU8P) | General theory of data, artificial intelligence and governance |  |  | Economics;  Science, technology and society |
| 72 | [(Djeffal, Siewert and Wurster, 2022)](https://paperpile.com/c/L9hHvg/yyaae) | Role of the state and responsibility in governing artificial intelligence: a comparative analysis of AI strategies | 22 countries; EU |  | Artificial intelligence; AI governance; policy instruments; state types; responsible research innovation; technology assessment |
| 73 | [(Xu, 2023)](https://paperpile.com/c/L9hHvg/ySMS) | ChatGPT opens a new door for bioinformatics |  |  | chatGPT, bioinformatics, alignment, literature mining, data analysis, method development, classroom, lifelong learning |
| 74 | [(Luo *et al.*, 2022)](https://paperpile.com/c/L9hHvg/30cA) | BioGPT: generative pre-trained transformer for biomedical text generation and mining |  |  | biomedical literature, generative pre-trained language model, text generation, text mining, problem solving protocol, BioGPT |
| 75 | [(Jin *et al.*, 2023)](https://paperpile.com/c/L9hHvg/HkFo) | GeneGPT: Augmenting Large Language Models with Domain Tools for Improved Access to Biomedical Information |  | preprint | GeneGPT, augmentation, LLMs, biomedical information, |
| 76 | [(Clusmann *et al.*, 2023)](https://paperpile.com/c/L9hHvg/WSqD) | The future landscape of large language models in medicine |  |  | Computational biology and bioinformatics,  Health services,  Medical research,  Public health, LLMs, Large language models (LLMs), OpenAI, ChatGPT, patient care, medical knowledge |
| 77 | [(Taylor *et al.*, 2023)](https://paperpile.com/c/L9hHvg/2xkt) | Intrinsic responsible innovation in a synthetic biology research project |  |  | Responsible Innovation, synthetic biology, interdisciplinarity, biocontainment, antibiotic resistance, lab escape, lab constructed genetic material |
| 78 | [(Tarasava, 2023)](https://paperpile.com/c/L9hHvg/1XoG) | How AI Is Transforming Synthetic Biology: Reaching Far Beyond Biopharma |  |  | Synthetic biology, biopharma, generative AI, protein structure prediction, molecular docking, enzyme design, protein design, gene expression optimization, workflow optimization, fermentation technology, fermentation scale-up, biosecurity |
| 79 | [(Aparicio, 2021)](https://paperpile.com/c/L9hHvg/Bwqw) | ‘That would break the containment’: the co-production of responsibility and safety-by-design in xenobiology |  |  | Technology of compliance, responsible research and innovation, ethics of synthetic biology, xenobiology, governance of emerging technologies, Responsible Research and Innovation (RRI), synthetic biology, public expectations, ethnography, interview data |
| 80 | [(Bianchini, 2023)](https://paperpile.com/c/L9hHvg/J3LP) | A New Definition of “Artificial” for Two Artificial Sciences |  |  | Artificial intelligence,  Synthetic biology,  Artificial-natural,  Biology,  Synthetic method, engineering, epistemology |
| 81 | [(Yeo and Selvarajoo, 2022)](https://paperpile.com/c/L9hHvg/vamg) | Machine learning alternative to systems biology should not solely depend on data |  |  | mechanistic modeling, machine learning, AI, systems biology, synthetic biology, metabolic engineering |
| 82 | [(Zampieri *et al.*, 2019)](https://paperpile.com/c/L9hHvg/nyJO) | Machine and deep learning meet genome-scale metabolic modeling |  |  | Machine learning, Metabolic networks, Machine learning algorithms, Cell metabolism, Principal component analysis,  Gene expression, Genomics, Metabolic pathways |
| 83 | [(Hassoun *et al.*, 2022)](https://paperpile.com/c/L9hHvg/McGr) | Artificial Intelligence for Biology |  |  | AI, biology, artificial intelligence, reintegration, statistics, computational scientists, vision, challenges, barriers, fragmentation of biology |
| 84 | [(Carbonell, Radivojevic and García Martín, 2019)](https://paperpile.com/c/L9hHvg/N20E) | Opportunities at the Intersection of Synthetic Biology, Machine Learning, and Automation |  | viewpoint | Algorithms,Biology,Genetics,Machine learning,Synthetic biology |
| 85 | [(Heyndrickx *et al.*, 2023)](https://paperpile.com/c/L9hHvg/iOaZ) | MELLODDY: Cross-pharma Federated Learning at Unprecedented Scale Unlocks Benefits in QSAR without Compromising Proprietary Information |  |  | Federated learning, MELLODY, pharma, crosspharma datasets, pharmacodynamics, pharmacokinetics, Quantitative Structure Activity Relationship (QSAR) models, machine learning, ML |
| 86 | [(Cubillos-Ruiz *et al.*, 2021)](https://paperpile.com/c/L9hHvg/L3qc) | Engineering living therapeutics with synthetic biology |  |  | Synthetic biology, living therapeutics, biologics, genetically engineered cells, synthetic gene circuits, biomarkers, biological sensing, engineered cell, therapeutic function, human diseases, diseases |
| 87 | [(Wong *et al.*, 2023)](https://paperpile.com/c/L9hHvg/ju8U) | Discovering small-molecule senolytics with deep neural networks |  |  | Ageing, Computer modelling,  Drug discovery, Machine learning, Senescence, bioinformatics, |
| 88 | [(Qureshi *et al.*, 2023)](https://paperpile.com/c/L9hHvg/1ciR) | AI in drug discovery and its clinical relevance |  |  | Artificial intelligence, Biotechnology, Graph neural networks, Molecule representation, Reinforcement learning, Drug discovery, Molecular dynamics simulation |
| **89** | [(Yan *et al.*, 2023)](https://paperpile.com/c/L9hHvg/58Nf) | Applications of synthetic biology in medical and pharmaceutical fields |  |  | Biotechnology, nanobiotechnology, biocircuits, standardized biological building blocks, synthetic biology, engineered cells, synthetic DNA circuits, biomedical applications, optogenetics, engineered live therapeutics, artificial intelligence, AI, |
| 90 | [(Patel *et al.*, 2009)](https://paperpile.com/c/L9hHvg/UC1d) | The coming of age of artificial intelligence in medicine |  |  | Artificial intelligence in medicine, history, Research challenges, Barriers to success, Sociocultural and cognitive factors, AI |
| 91 | [(Damiano, Kuruma and Stano, 2016)](https://paperpile.com/c/L9hHvg/7nOu) | What can synthetic biology offer to artificial intelligence (and vice versa)? |  | Editorial | Synthetic biology, AI |
| 92 | [(Zhao, 2022)](https://paperpile.com/c/L9hHvg/PtLy) | Synthetic Biology 2.0: The Dawn of a New Era |  | Editorial | Synthetic biology, AI, automation, bioeconomy |
| 93 | [(El Karoui, Hoyos-Flight and Fletcher, 2019)](https://paperpile.com/c/L9hHvg/sVRY) | Future Trends in Synthetic Biology—A Report |  |  | synthetic biology, biosystem, future trends and developments, biodesign automation, responsible research and innovation (RRI) |
| 94 | [(Voigt, 2020)](https://paperpile.com/c/L9hHvg/YnpI) | Synthetic biology 2020–2030: six commercially-available products that are changing our world |  |  | Synthetic biology, transformation, future, futurist, foresight, products, fermentation, systems, cells as products, engineered biology |
| 95 | [(Radivojević *et al.*, 2020)](https://paperpile.com/c/L9hHvg/tV0j) | A machine learning Automated Recommendation Tool for synthetic biology |  |  | Bayesian inference,  Machine learning,  Metabolic engineering,  Synthetic biology, Automated Recommendation Tool (ART) |
| 96 | [(Zhang *et al.*, 2023)](https://paperpile.com/c/L9hHvg/Gffq) | Enabling technology and core theory of synthetic biology |  |  | Quantitative synthetic biology, AI, biofoundries, DNA storage, gene editing |
| 97 | [(Watters and Lemanski, 2023)](https://paperpile.com/c/L9hHvg/LxjR) | Universal skepticism of ChatGPT: a review of early literature on chat generative pre-trained transformer |  |  | ChatGPT, large language model (LLM), transformer, GPT, disruptive technology, artificial intelligence, AI, disciplines, healthcare |
| 98 | [(Lubiana *et al.*, 2023)](https://paperpile.com/c/L9hHvg/F2JP) | Ten quick tips for harnessing the power of ChatGPT in computational biology |  |  | chatGPT, computational biology, tips, tool, cleanup data, engineer prompts, summarization, AI tools |
| 99 | [(Ray, 2023)](https://paperpile.com/c/L9hHvg/ZzuS) | ChatGPT: A comprehensive review on background, applications, key challenges, bias, ethics, limitations and future scope |  |  | ChatGPT, Language modelGPT-3.5, Generative AI, Conversational AI, Context understanding, Natural language processing |
| 100 | [(Harrer, 2023)](https://paperpile.com/c/L9hHvg/aQ9u) | Attention is not all you need: the complicated case of ethically using large language models in healthcare and medicine |  |  | Generative artificial intelligence,  Large language models,  Foundation models,  AI ethics,  Augmented human intelligence,  Information management,  AI trustworthiness |
| 101 | [(Morris, 2023)](https://paperpile.com/c/L9hHvg/uUxR) | Scientists’ Perspectives on the Potential for  Generative AI in their Fields |  | preprint | Generative AI, science, scientists, scientific findings, augmentation, concerns, literature review, coding, discovery, communication, mitigation, negative side-effects, intentional misappropriation |
| 102 | [(Kather *et al.*, 2022)](https://paperpile.com/c/L9hHvg/Y6Ck) | Medical domain knowledge in domain-agnostic generative AI |  |  | Biomedical engineering,  Data integration,  Machine learning |
| 103 | [(Xiangxiang Zeng *et al.*, 2022)](https://paperpile.com/c/L9hHvg/z8BD) | Deep generative molecular design reshapes drug discovery |  |  | Generative models, AI, drug discovery, drug development challenges, multimodal deep generative models |
| 104 | [(Bhardwaj, Kishore and Pandey, 2022)](https://paperpile.com/c/L9hHvg/5FxZ) | Artificial Intelligence in Biological Sciences |  |  | artificial intelligence, biotechnology, agriculture, medicine, crop yield, life science, metabolic pathways, bio-based industrial setup, bio-based industry, bioengineering |
| 105 | [(Topol, 2019)](https://paperpile.com/c/L9hHvg/sT3j) | High-performance medicine: the convergence of human and artificial intelligence |  |  | Health care, machine learning, AI, human intelligence, artificial intelligence, AI in life science, genomics, multi-omics datasets, |
| 106 | [(Camacho *et al.*, 2018)](https://paperpile.com/c/L9hHvg/odAg) | Next-Generation Machine Learning for Biological Networks |  |  | Machine learning, deep learning, systems biology,  synthetic biology, network biology, neural networks, biological networks, microbiome research, disease biology, gene circuits, synthetic gene circuits, synthetic circuit, biomedical applications, fundamental design principles, synthetic gene networks |
| 107 | [(Mökander *et al.*, 2022)](https://paperpile.com/c/L9hHvg/Z9FJ) | Challenges and best practices in corporate AI governance: Lessons from the biopharmaceutical industry |  |  | artificial intelligence, AstraZeneca, case study, ethics, governance, implementation, lessons learned, practice |
| 108 | [(Edwards, 2014)](https://paperpile.com/c/L9hHvg/rHKu) | Taking Stock of Security Concerns Related to Synthetic Biology in an Age of Responsible Innovation |  |  | synthetic biology, biosafety, biosecurity, ethics, dual-use, public health, policy, ethical, legal, and social issue (ELSI), responsible innovation , dual-use research of concern (DURC) |
| 109 | [(Lev, 2019)](https://paperpile.com/c/L9hHvg/tZwu) | Regulating dual-use research: Lessons from Israel and the United States |  |  | Dual-use, dual-use research of concern (DURC). Legislation, Israel, USA, China, policy |
| 110 | [(Xiaomei Zeng *et al.*, 2022)](https://paperpile.com/c/L9hHvg/0OS1) | Regulation and management of the biosecurity for synthetic biology |  |  | Synthetic biology, Biosecurity risk, Regulation and management, Source, Suggestion, synbio, biosafety, biosecurity, GMO, China, risk assessment, standard, biosafety standards, labs |
| 111 | [(Ienca and Vayena, 2018)](https://paperpile.com/c/L9hHvg/OKor) | Dual use in the 21st century: emerging risks and global governance |  |  | dual-use research of concern (DURC), global governance, decentralized governance architectures, bottom-up strategies,S WHO |
| 112 | [(Palmer, Fukuyama and Relman, 2015)](https://paperpile.com/c/L9hHvg/isuj) | A more systematic approach to biological risk |  |  | Biological risk, life science, Asilomar, U.S. biosafety, policy, regulation, gain-of-function, viral pathogens, gene drives, security risks |
| 113 | [(Pannu *et al.*, 2022)](https://paperpile.com/c/L9hHvg/PpYg) | Strengthen oversight of risky research on pathogens |  |  | Pathogens, oversight, governance, policy reset, convergence, DURC, dual-use research of concern (DURC) |
| 114 | [(Cho and Relman, 2010)](https://paperpile.com/c/L9hHvg/tdIt) | Synthetic “Life,” Ethics, National Security, and Public Discourse |  |  | Synthetic life, artificial life, national security, public discourse, synthetic biology, risks, U.S. National Science Advisory Board for Biosecurity (NSABB) |
| 115 | [(Sun *et al.*, 2022)](https://paperpile.com/c/L9hHvg/zNjW) | Challenges and recent progress in the governance of biosecurity risks in the era of synthetic biology |  |  | Synthetic biology, Biosecurity, Risk management, Governance systems, International collaboration, aggravation of species with complex gene modifications, threats to species diversity, abuse of biological weapons, laboratory leaks, and man-made mutations, China |
| 116 | [(J. Sandbrink, 2023)](https://paperpile.com/c/L9hHvg/lV6s) | Panoptic dual-use management: preventing deliberate pandemics in an age of synthetic biology and artificial intelligence |  | Ph.D thesis | Dual-use, pandemics, synthetic biology, artificial intelligence, LLMs, gene therapy, wildlife virus discovery, viral engineering for vaccine design, viral engineering for gene therapy, management of dual-use virological research, mitigation regimes, tiered dual-use scores |
| 117 | [(Evans, , 2022)](https://paperpile.com/c/L9hHvg/3Caj) | When All Research Is Dual Use |  |  | Biosecurity, threats, social context, machine intelligence, AI, biological weapons, compliance, dual use, dual use research, security governance, pathogens, curiosity-based system |
| 118 | [(Tang *et al.*, 2023)](https://paperpile.com/c/L9hHvg/LSBf) | Synthetic biology and governance research in China: a 40-year evolution |  |  | Synthetic biology,  Biosafety,  Biosecurity,  Structural topic model,  Governance of emerging technologies, synbio, China, safety research, policy implications, synbio safety |
| **119** | [(Pei, Garfinkel and Schmidt, 2022)](https://paperpile.com/c/L9hHvg/xqMy) | Bottlenecks and opportunities for synthetic biology biosafety standards |  |  | Bioremediation,  Environmental biotechnology,  Synthetic biology, standards, biosafety, standardization, biosafety standardization, biosecurity, biocontainment |
| 120 | [(Ou and Guo, 2023)](https://paperpile.com/c/L9hHvg/PZPB) | Safety risks and ethical governance of biomedical applications of synthetic biology |  |  | synthetic biology, participant safety, biosafety risks, biosecurity risks, ethical governance, public policy, biomedicine, China |
| 121 | [(Millett *et al.*, 2020)](https://paperpile.com/c/L9hHvg/5nUb) | The synthetic-biology challenges for biosecurity: examples from iGEM |  |  | Biosecurity, synthetic biology, education, Australia Group, export controls, dual use, biological engineering, International Genetically Engineered Machine competition (iGEM), iGEM, biosecurity procedures |
| 122 | [(Trump *et al.*, 2020)](https://paperpile.com/c/L9hHvg/hPvy) | Building biosecurity for synthetic biology |  |  | Biosecurity, synthetic biology, bioterrorism, non-state actors, potential misusers, non-state organizations, bioweapons, anthrax, botulinum toxin, oversight, security |
| 123 | [(Gronvall, 2018)](https://paperpile.com/c/L9hHvg/OQc3) | Safety, security, and serving the public interest in synthetic biology |  |  | Synthetic Biology,  Technical Expertise Required,  National Science Advisory Board For Biosecurity (NSABB),  Biological Weapons Development,  Hazardous Biological Materials, biosafety, toxins, policy, safety, security, public interest |
| 124 | [(Schaefer *et al.*, 2023)](https://paperpile.com/c/L9hHvg/qLY0) | Large language models are universal biomedical simulators |  | preprint | large language models (LLMs), GPT-4, SimulateGPT, biomedical applications, biological simulators, complex living systems |
| 125 | [(Nguyen *et al.*, 2023)](https://paperpile.com/c/L9hHvg/GkhL) | Bioengineering of bacteria for cancer immunotherapy |  |  | Cancer, Cancer microenvironment,  Cancer prevention, synbio, bioengineering, synthetic biology, immunotherapy, drug development, BCiT, genetic engineering, genetic circuits, bacteria-based cancer immunotherapy (BCiT), bacteria, S. typhimurium, E. coli |
| 126 | [(Millett and Alexanian, 2021)](https://paperpile.com/c/L9hHvg/1A46) | Implementing adaptive risk management for synthetic biology: Lessons from iGEM's safety and security programme |  |  | adaptive risk management, biosafety, biosecurity, emerging technology, iGEM, reflexive risk management, risk management, synthetic biology |
| 125 | [(Beeckman and Rüdelsheim, 2020)](https://paperpile.com/c/L9hHvg/2zNU) | Biosafety and Biosecurity in Containment: A Regulatory Overview |  |  | biosafety, biosecurity, biological material, biological agent, containment, regulations, (bio)risk assessment |
| 126 | [(Linkov, Trump, Poinsatte-Jones, *et al.*, 2018)](https://paperpile.com/c/L9hHvg/9YBI) | Governance Strategies for a Sustainable Digital World |  |  | digital revolution; sustainability challenges; governance strategies, laissez-faire, industry-driven approach, precautionary approach, preemptive strategy, stewardship |
| 127 | [(Keiper and Atanassova, 2020)](https://paperpile.com/c/L9hHvg/aIQP) | Regulation of Synthetic Biology: Developments Under the Convention on Biological Diversity and Its Protocols |  |  | synthetic biology, living modified organisms, Cartagena Protocol on Biosafety, Nagoya Protocol on Access and Benefit Sharing, risk assessment, gene drives, digital sequence information, biotechnology regulation |
| 128 | [(Tebeje, Tadesse and Mengesha, 2021)](https://paperpile.com/c/L9hHvg/wq0z) | Synthetic bio/techno/logy and its application |  |  | Synthetic genomics, synthetic biology, genome minimization, bottom-up, top-dow |
| 129 | [(Perrakis and Sixma, 2021)](https://paperpile.com/c/L9hHvg/DRhp) | AI revolutions in biology |  |  | AI revolutions, biology, protein folding, AlphaFold, structural biology, experimental structural biology, protein fold predictions, DeepMind, European Bioinformatics Institute (EBI), RoseTTAfold, deep learning, AI, experiments, AI dependence, AI in science, drug discovery |
| 130 | [(Seydel, 2023)](https://paperpile.com/c/L9hHvg/Mvoe) | DNA writing technologies moving toward synthetic genomes |  | News | DNA synthesis, synthetic genomes, biosecurity, biomanufacturing, designer genes, regulation, business models, design-build-test cycle |
| **131** | [(Hoffmann *et al.*, 2023)](https://paperpile.com/c/L9hHvg/JGH6) | Safety by design: Biosafety and biosecurity in the age of synthetic genomics |  |  | Biological sciences, Systems biology, Genomics, safety by design, biosafety, biosecurity, synthetic genomics, bioeconomy, regulatory frameworks, technology frameworks, risks, technology readiness levels, synthetic DNA, synthetic DNA of concerns, sequence screening, genetic biocontainment systems, environmental proliferation, DNA synthesis, synthetic yeast, synthetic eukaryotic genome, designer genomes, biofoundries, DIY BIO, International Gene Synthesis Consortium (IGSC), environmental metagenomics, suicide genes, biocontainment |
| **132** | [(Rudolph *et al.*, 2023)](https://paperpile.com/c/L9hHvg/C4LS) | Strategies to identify and edit improvements in synthetic genome segments episomally |  |  | Synthetic biology, bioengineering, synthetic genome, gene editing, bacterial artificial chromosomes (BACs), multiplex automated genome engineering (MAGE) |
| **133** | [(Hınçer *et al.*, 2023)](https://paperpile.com/c/L9hHvg/I7nT) | Making the Next Generation of Therapeutics: mRNA Meets Synthetic Biology |  |  | mRNA-based therapeutics, mRNA vaccines, synthetic biology, self-assembled nanoparticles, logic gates |
| 134 | [(Beardall, Stan and Dunlop, 2022)](https://paperpile.com/c/L9hHvg/S36y) | Deep Learning Concepts and Applications for Synthetic Biology |  |  | mRNA-based therapeutics, mRNA vaccines, synthetic biology, self-assembled nanoparticles, logic gates |
| 135 | [(Belda *et al.*, 2021)](https://paperpile.com/c/L9hHvg/khRJ) | Seeding the idea of encapsulating a representative synthetic metagenome in a single yeast cell |  |  | Microbial ecology,  Synthetic biology, designer yeasts, |
| 136 | [(Dixon and Pretorius, 2020)](https://paperpile.com/c/L9hHvg/dCcH) | Drawing on the Past to Shape the Future of Synthetic Yeast Research |  |  | Synthetic yeast, biodesign; biodiversity; biofoundry; consilience; engineering biology; fermentation; scientific method; synthetic genomics; timeline; yeast, foresight, history of science, world’ greatest challenges |
| 137 | [(Wani *et al.*, 2022)](https://paperpile.com/c/L9hHvg/WGsE) | Metagenomics and artificial intelligence in the context of human health |  |  | Microbiome, Metagenomics, Artificial intelligence, Human health, Diseases |
| 138 | [(Wang *et al.*, 2023)](https://paperpile.com/c/L9hHvg/IS5A) | Ethical and social insights into synthetic biology: predicting research fronts in the post-COVID-19 era |  |  | synthetic biology, social sciences, bioethical concerns, playing God, biosecurity, biosafety, governance, stakeholders, |
| 139 | [(Hammang, 2023)](https://paperpile.com/c/L9hHvg/mx9D) | Troubleshooting: The Automation of Synthetic Biology and the Labor of Technological Futures |  |  | Automation, synthetic biology, work of the future, future of work, technological futures, Science and technology studies, STS, skilled work, troubleshooting, temporality, laboratory work, interviews, laboratory technicians, ethnography, efficiency, digital infrastructure, robotic infrastructure, workflow, innovation, quality control, maintenance |
| 140 | [(Zhao *et al.*, 2023)](https://paperpile.com/c/L9hHvg/OxsG) | Synthetic biology-inspired cell engineering in diagnosis, treatment, and drug development |  |  | Biomarkers,  Cancer,  Drug delivery,  Genetic engineering, machine learning, computational biology |
| 141 | [(Pansera *et al.*, 2020)](https://paperpile.com/c/L9hHvg/DcZn) | Embedding responsible innovation within synthetic biology research and innovation: insights from a UK multi-disciplinary research centre |  |  | Responsible research and innovation, RI, research governance, synthetic biology, public engagement, UK, reflexivity, RI institutionalization |
| 142 | [(Shapira, Kwon and Youtie, 2017)](https://paperpile.com/c/L9hHvg/HQpy) | Tracking the emergence of synthetic biology |  |  | Emerging technology, Synthetic biology, Bibliometric analysis, Search strategy, Map of science, Research sponsors |
| 143 | [(Nylund *et al.*, 2022)](https://paperpile.com/c/L9hHvg/1ayF) | The emergence of entrepreneurial ecosystems based on enabling technologies: Evidence from synthetic biology |  |  | Entrepreneurial ecosystems, Ecosystem emergence, Complex adaptive systems, Enabling technologies, Synthetic biology |
| 144 | [(Hamlyn, 2022)](https://paperpile.com/c/L9hHvg/TsCT) | Synthesize this: integrating innovation governance and EU regulation of synthetic biology |  |  | Synthetic biology, innovation governance, EU, science and technology studies (STS), Responsible innovation (RI), synbio , governance continuum, participatory governance |
| 145 | [(Lee and George, 2023)](https://paperpile.com/c/L9hHvg/CaP1) | Future worldbuilding with synthetic biology: A case study in interdisciplinary scenario visualization |  |  | Biodesign, Design fiction, Futures studies, Scenario visualization, worldbuilding, plant metabolites, microalgae, scenario |
| 146 | [(Kirksey, 2021)](https://paperpile.com/c/L9hHvg/sezo) | Living Machines Go Wild |  |  | Anthropology, synthetic biology, iGEM, students, genetic engineering tools, competition |
| 147 | [(Fulvi and Wodak, 2023)](https://paperpile.com/c/L9hHvg/XiIA) | Gambling on unknown unknowns: Risk ethics for a climate change technofix |  |  | Risk ethics, climate change, technofix, Negative Emissions Technologies (NETs), Synthetic Biology, IPCC, uncertainty |
| 148 | [(Esquivel-Sada, 2022)](https://paperpile.com/c/L9hHvg/ihM5) | Responsible intellectual property rights? Untangling open-source biotech adherence to intellectual property rights through DIYbio |  |  | Open-source biotechnology, Intellectual property, Bio-hacking, DIYbio, Synthetic biology, Life patenting, anti-commons crisis, Do-It-Yourself biology, IP rights, dual-use device, dual-use systems |
| 149 | [(Wickberg, 2021)](https://paperpile.com/c/L9hHvg/QzNg) | Strange Natures: Conservation in the Era of Synthetic Biology |  | Book review | Synthetic biology, conservation, book review |
| **150** | [(Ribeiro *et al.*, 2023)](https://paperpile.com/c/L9hHvg/poKL) | The digitalisation paradox of everyday scientific labour: How mundane knowledge work is amplified and diversified in the biosciences |  |  | Automation, Digitalization, Synthetic biology, Practice, Work, Labor |
| 151 | [(Szocik *et al.*, 2021)](https://paperpile.com/c/L9hHvg/UIql) | Future space missions and human enhancement: Medical and ethical challenges |  |  | Space missions, Space settlement, Human enhancement, Gene editing, CRISPR, Synthetic biology, Bioethics, implants, ethics |
| 152 | [(Holland *et al.*, 2024)](https://paperpile.com/c/L9hHvg/U95f) | Innovation intermediaries at the convergence of digital technologies, sustainability, and governance: A case study of AI-enabled engineering biology |  |  | Innovation intermediaries, Responsible innovation, Technological convergence, sustainability, governance, AI, AI-enabled engineering, AI, convergence, biomanufacturing, bioeconomy, synthetic biology, engineering biology, applications, digital-enabled transformation, design-build-test systems |
| 153 | [(Lawson *et al.*, 2021)](https://paperpile.com/c/L9hHvg/ApSV) | Machine learning for metabolic engineering: A review |  |  | Machine Learning, Metabolic Engineering, Synthetic Biology, Deep Learning, practitioners, engineers, scale-up, future perspectives, metabolic engineers, biochemists, microbiologists, computer scientists, electrical engineers, chemical engineers, mathematicians, statisticians, physicists, AI |
| 154 | [(Watkins *et al.*, 2023)](https://paperpile.com/c/L9hHvg/81T7) | Public biofoundries as innovation intermediaries: the integration of translation, sustainability, and responsibility |  |  | Innovation intermediaries,  Public biofoundries,  Engineering biology,  Translation,  Sustainability,  Responsibility |
| 155 | [(Vinke, Rais and Millett, 2022)](https://paperpile.com/c/L9hHvg/eBoD) | The Dual-Use Education Gap: Awareness and Education of Life Science Researchers on Nonpathogen-Related Dual-Use Research |  |  | Dual-usescience, Biosecurity, Bioethics, Molecular biology, synthetic biology, iGEM, self-regulatory approach |
| 156 | [(Mourby *et al.*, 2022)](https://paperpile.com/c/L9hHvg/RbNN) | Biomodifying the ‘natural’: from Adaptive Regulation to Adaptive Societal Governance |  |  | Biomodifying technologies, regulation, adaptive regulation, societal governance, governance, gene editing, induced pluripotent stem cells, bioprinting, applications, lab-grown meat, pest control, medicine, assessment schemes, regulatory flexibility, stakeholder engagement, biomodification, global observatory model, biotechnology, legislative flexibility |
| 157 | [(Gisselsson, 2022)](https://paperpile.com/c/L9hHvg/9zlZ) | Next-Generation Biowarfare: Small in Scale, Sensational in Nature? |  |  | Biowarfare, synthetic biology |
| 158 | [(Meissner *et al.*, 2021)](https://paperpile.com/c/L9hHvg/Kdmd) | The rise of do-it-yourself (DiY) laboratories: Implications for science, technology, and innovation (STI) policy |  |  | DiY laboratories, STI policy, science, technology, and innovation (STI) policy |
| 159 | [(Dixon, 2023)](https://paperpile.com/c/L9hHvg/ZXEY) | The bioinformational dilemma: where bioinformational diplomacy meets cyberbiosecurity |  |  | Bioinformational diplomacy, cyberbiosecurity, COVID-19, bioinformational secrecy, security dilemma, bioinformational dilemma, bioinformation, security, national security, diplomacy, global public health, emergencies, global public health emergencies, |
| 160 | [(Bauer and Bogner, 2020)](https://paperpile.com/c/L9hHvg/0bV4) | Let's (not) talk about synthetic biology: Framing an emerging technology in public and stakeholder dialogues |  |  | Synthetic biology, technoscience, biotechnology debates, media frames, social progress, innovation, ethics, EU, EU FP7, Synergene, public policy, STS, risk, risk frame, biosafety, biosecurity risks, public dialogue, social engagement, technology policy, technology optimism, meta-frames, DIY-biology, NEST-ethics, synbio, Responsible Research and Innovation (RRI), bio-capitalism, social progress frame, science frame, ethics frame |
| 161 | [(Fantine, Margis, R., Talamini, E.l and Dewes, 2023)](https://paperpile.com/c/L9hHvg/e0z2) | Trends in Synthetic Biology in the Bioeconomy of Non-Food-Competing Biofuels |  |  | microbial production; bioethanol; biodiesel; SynB; food security; information science; scientometry |
| 162 | [(Mialon *et al.*, 2023)](https://paperpile.com/c/L9hHvg/IUsY) | GAIA: a benchmark for General AI Assistants |  | preprint | GAIA, AI assistants, GPT-4, AI benchmarks, fundamental abilities, reasoning, multi-modality handling, web browsing, tool-use proficiency, AGI, Artificial General Intelligence, LLMs |
| 163 | [(Baker, 2017)](https://paperpile.com/c/L9hHvg/qJEU) | Synthetic Biology and the Marketplace: Building the new bioeconomy |  |  | Bioeconomy, synthetic biology, marketplace, DNA synthesis, Twist Bioscience, foundry, biofoundry, Ginkgo Bioworks, iGEM |
| 164 | [(Hanczyc, 2020)](https://paperpile.com/c/L9hHvg/iHje) | Engineering Life: A Review of Synthetic Biology |  | Review | Synthetic biology, genetic engineering, metabolic engineering, artificial cell, model-driven design, cell-free approaches, MAGE, feedback control, optimization, system control, modularity, standardization |
| 165 | [(Garner, 2021)](https://paperpile.com/c/L9hHvg/nrRC) | Principles of synthetic biology |  |  | metabolic engineering, synthetic cells, regulatory circuits, synthetic genome, Design–Build–Test–Learn cycle, standardization, biomaterials, plug-and-play, off-the-shelf bioparts, bioparts |
| 166 | [(Frow, 2020)](https://paperpile.com/c/L9hHvg/hICl) | From “Experiments of Concern” to “Groups of Concern”: Constructing and Containing Citizens in Synthetic Biology |  |  | Synthetic biology, citizens, groups of concern, experiments of concern, governance, USA, UK, DNA technology, recombinant DNA technology, self-governance mechanisms, sociotechnical imaginaries, risks, national approaches, Asilomar |
| 167 | [(Freemont, 2019)](https://paperpile.com/c/L9hHvg/6f9q) | Synthetic biology industry: data-driven design is creating new opportunities in biotechnology |  |  | biotechnology, industry, synthetic biology, startups, technology stack, open technology sharing, democratization, multinational corporations, technology monopolies, manufacturing revolution, biotechnology products, living therapies, synthetic biology market, biotechnology industry, biopharma industry |
| 168 | [(Gomez-Hinostroza *et al.*, 2023)](https://paperpile.com/c/L9hHvg/0EmX) | Current landscape and future directions of synthetic biology in South America |  |  | SynBio, South America, DIY, Latin America, TECNOx, Open science |
| 169 | [(David *et al.*, 2021)](https://paperpile.com/c/L9hHvg/uOZk) | A Perspective on Synthetic Biology in Drug Discovery and Development—Current Impact and Future Opportunities |  |  | Synthetic biology, drug discovery, impact, DNA synthesis, data science, workflows, AI, machine learning |
| 170 | [(Marris and Calvert, 2020)](https://paperpile.com/c/L9hHvg/GZlx) | Science and Technology Studies in Policy: The UK Synthetic Biology Roadmap |  |  | STS, roadmap, UK, framing, responsible research and innovation (RRI), science and society, STS in policy, STS researchers, policy process, roadmapping, technological innovation, economic progress |
| 171 | [(Vallero Daniel A. and Gunsch Claudia K., 2020)](https://paperpile.com/c/L9hHvg/O1Yp) | Applications and Implications of Emerging Biotechnologies in Environmental Engineering |  |  | Synthetic biology, emerging biotechnologies, environmental engineering, remediation, waste treatment, risks, complexities, risk perception, risk assessment, ethics, engineering, environmental engineers, preventive approach |
| 172 | [(Zarnack and Eyras, 2023)](https://paperpile.com/c/L9hHvg/8aRQ) | ‘Artificial intelligence and machine learning in RNA biology’ |  |  | Algorithms, AI, biology, machine learning, RNA biology, bioinformatics, RNA structure prediction |
| 173 | [(Jatain *et al.*, 2021)](https://paperpile.com/c/L9hHvg/OUKs) | Synthetic biology potential for carbon sequestration into biocommodities |  |  | CO2 fixation pathway, CO2 sequestration, Metabolic engineering, Synthetic biology, System biology, Bioproducts, global warming, renewable energy source, microbial cell factories, biocommodities, carbon sequestration |
| 174 | [(Matsuyama and Suzuki, 2019)](https://paperpile.com/c/L9hHvg/8KTT) | Systems and Synthetic microRNA Biology: From Biogenesis to Disease Pathogenesis |  |  | microRNA, RNAi, Drosha, Argonaute, biogenesis, super-enhancer, disease pathogenesis, synthetic biology |
| 175 | [(Naderi Yeganeh *et al.*, 2023)](https://paperpile.com/c/L9hHvg/bxtm) | PanomiR: a systems biology framework for analysis of multi-pathway targeting by miRNAs |  |  | microRNA, miRNA regulation, pathways, biological networks, systems biology, miRNA prioritization, pathway analysis, bioinformatics, computational biology, statistical models |
| 176 | [(van Doren *et al.*, 2022)](https://paperpile.com/c/L9hHvg/uadh) | The external commercialisation of technology in emerging domains – the antecedents, consequences, and dimensions of desorptive capacity |  |  | Desorptive capacity, exploration & exploitation, management innovation, emerging technology domains, synthetic biology, commercial risks, technology commercialization, synthetic biology organizations, External technology commercialisation (ETC), positive network externalities |
| 177 | [(Banda and Huzair, 2021)](https://paperpile.com/c/L9hHvg/j7I2) | Introduction to special issue: innovation/governance interactions in the bioeconomy |  | Introduction | Bioeconomy, governance, synthetic biology |
| 178 | [(Tait and Wield, 2021)](https://paperpile.com/c/L9hHvg/SG4W) | Policy support for disruptive innovation in the life sciences |  |  | Science & technology and innovation policy studies, foresight, radical/disruptive innovation, industrial biotechnology |
| 179 | [(Ahmed and Harrison, 2021)](https://paperpile.com/c/L9hHvg/sg7y) | Challenges and competencies of entrepreneurial leaders in driving innovation at DIY laboratories |  |  | Entrepreneurship, leadership, entrepreneurial leadership, DIY innovation, laboratories, synthetic biology, machine learning, office automation, technological competence |
| 180 | [(Damoah and Botchie, 2021)](https://paperpile.com/c/L9hHvg/GRWG) | Do-It-Yourself (DIY) laboratories and science, technology, and innovation (STI): trends, implications and future research |  |  | Do-It-Yourself laboratory (DIY), Science technology and innovation (STI), systematic literature review, DIY activities |
| 181 | [(Dzandu and Pathak, 2021)](https://paperpile.com/c/L9hHvg/U6LM) | Diy laboratories, their practices, and challenges – a systematic literature review |  |  | Do-it-yourself, DiY laboratories, innovation diffusion, digital innovation |
| 182 | [(Liedong and Sarpong, 2021)](https://paperpile.com/c/L9hHvg/gEda) | Taking stock and charting the future: the management and implications of DIY laboratories for innovation and society |  |  | Innovation studies, risk analysis and technology, technology & innovation studies, public understanding of science and technology |
| 183 | [(Huzair, 2021)](https://paperpile.com/c/L9hHvg/cYOd) | Risk and regulatory culture: governing recombinant DNA technology in the UK from 1970–1980 |  |  | Regulation, biotechnology, DNA, UK, recombinant DNA, laboratory work, laboratory safety, workers rights, epistemic cultures, regulatory space, deregulation |
| 184 | [(Boyd and Wilson, 2020)](https://paperpile.com/c/L9hHvg/R43V) | Existential Risks to Humanity Should Concern International Policymakers and More Could Be Done in Considering Them at the International Governance Level |  |  | Artificial intelligence; catastrophic risk; existential risk; nuclear war; synthetic biology; United Nations |
| 185 | [(Thiele, 2020)](https://paperpile.com/c/L9hHvg/TA2b) | Nature 4.0: Assisted Evolution, De-extinction, and Ecological Restoration Technologies |  |  | Assisted evolution, ecological restoration, synthetic biology, Nature 4.0, re-create nature, biodiversity, cultural risks, ecological risks, risks, de-extinct species, environmentalists, conservationists, ecological restoration technologies |
| 186 | [(Lockhart, Marvin and While, 2023)](https://paperpile.com/c/L9hHvg/zg0o) | Towards new ecologies of automation: Robotics and the re-engineering of nature |  |  | Automation, ecology, robotics, reengineering of nature, synthetic biology, climate breakdown, anthropocene, operational ecologies, critical geography, automation studies, political ecology |
| 187 | [(Merchant *et al.*, 2023)](https://paperpile.com/c/L9hHvg/bRUB) | Scaling deep learning for materials discovery |  |  | Deep learning, materials discovery, applications |
| 188 | [(Tang *et al.*, 2020)](https://paperpile.com/c/L9hHvg/anXZ) | Materials design by synthetic biology |  |  | Synthetic biology, materials design, genetic circuits, smart living materials, living sensors, therapeutics, electronics, programmable living materials, biomimicry, biomaterials, AI, machine learning |
| 189 | [(Mateu-Sanz *et al.*, 2023)](https://paperpile.com/c/L9hHvg/nILc) | Redefining biomaterial biocompatibility: challenges for artificial intelligence and text mining |  |  | Biomaterial, biocompatibility, databases, artificial intelligence, data mining, natural language processing, international organization for standardization (ISO) |
| 190 | [(Burgos-Morales *et al.*, 2021)](https://paperpile.com/c/L9hHvg/c9x1) | Synthetic biology as driver for the biologization of materials sciences |  |  | Interactive materials, Engineered living materials, Metabolic engineering, Cell encapsulation, Nanomaterials, Smart materials, Stimulus-responsive materials, AI, machine learning, synthetic biology, materials sciences |
| 191 | [(Scheper *et al.*, 2021)](https://paperpile.com/c/L9hHvg/VdDv) | Digitalization and Bioprocessing: Promises and Challenges |  | Book chapter | Digitalization, Digital twins  FDA, QbD, Regulatory considerations, bioprocessing, biotech, synthetic biology, biopharmaceuticals |
| 192 | [(Zhu *et al.*, 2023)](https://paperpile.com/c/L9hHvg/nBMj) | Current advances of biocontainment strategy in synthetic biology |  |  | Genetically modified organisms (GMOs), Biocontainment, Biosecurity, Synthetic biology, Molecular biology, Biotechnology |
| 193 | [(Asin-Garcia *et al.*, 2020)](https://paperpile.com/c/L9hHvg/qhZ3) | Genetic Safeguards for Safety-by-design: So Close Yet So Far |  |  | Safety-by-designs, synthetic biology, genetic safeguards, biocontainment, biosafety, auxotrophy, semantic containment, combinatorial systems |
| 194 | [(Schmidt and de Lorenzo, 2016)](https://paperpile.com/c/L9hHvg/rV5C) | Synthetic bugs on the loose: containment options for deeply engineered (micro)organisms |  |  | Synthetic biology, engineered microorganisms, synthetic bugs, xenobiology, certainty-of-containment, gene drive, standardized metrics |
| 195 | [(Moon, 2023)](https://paperpile.com/c/L9hHvg/g3IO) | EBRC: Enhancing bioeconomy through research and communication |  |  | Executive order, Biomanufacturing, Biotechnology, Next generation, Disruptive research, Diversity, equity and inclusion, International collaboration, Biosecurity, biosafety and bioethics, Data sharing, Global issue, GMO, CRISPR, USA, Dual Use Research of Concern (DURC), antibiotic resistance genes (ARGs) , genetically engineered microbes (GEMs), user-friendly interfaces, DEI |
| 196 | [(Bohua *et al.*, 2023)](https://paperpile.com/c/L9hHvg/JOgZ) | Ethical framework on risk governance of synthetic biology |  |  | Synthetic biology, Ethical framework, Biosafety, Precautionary principle, Environmental risks |
| 197 | [(Lee and Kim, 2023)](https://paperpile.com/c/L9hHvg/RstJ) | Cell-Free Synthetic Biology: Navigating the New Frontiers of Biomanufacturing and Biological Engineering |  |  | synthetic biology, cell-free synthetic biology, systems biology, protein synthesis, metabolic engineering, biomaterials, machine learning, AI |
| 198 | [(Tinafar, Jaenes and Pardee, 2019)](https://paperpile.com/c/L9hHvg/iVDm) | Synthetic Biology Goes Cell-Free |  |  | Cell-free systems (CFS), synthetic biology, AI rational design, genetically programmable tools, in vitro platforms, centralized production, high-value commodities, unexpected outcomes, creative outcomes, nano-scale materials, engineered enzymes, machine learning |
| 199 | [(Dixon *et al.*, 2022)](https://paperpile.com/c/L9hHvg/5qcR) | A global forum on synthetic biology: the need for international engagement |  |  | Synthetic biology, molecular engineering, policy. Flourishing, minimising the downsides, global forum, COVID-19, dual-use, governance |
| 200 | [(de Lorenzo, Krasnogor and Schmidt, 2021)](https://paperpile.com/c/L9hHvg/9hjw) | For the sake of the Bioeconomy: define what a Synthetic Biology Chassis is! |  |  | GMOs, recombinant DNA, genetic implant, traceability, containment, barcoding, digital twins, watermarking, DNA steganography, Pseudomonas putida, synbio chassis, generally regarded as safe (GRAS), environmental risk assessment (ERA) |
| 201 | [(Rennings, Burgsmüller and Bröring, 2023)](https://paperpile.com/c/L9hHvg/u2Qe) | Convergence towards a digitalized bioeconomy—Exploring cross-industry merger and acquisition activities between the bioeconomy and the digital economy |  |  | Bioeconomy, M&A, digital economy, convergence literature, coupling transition theory |
| 202 | [(Bröring, Laibach and Wustmans, 2020)](https://paperpile.com/c/L9hHvg/y7g3) | Innovation types in the bioeconomy |  |  | Innovation types, Bioeconomy, Circular economy, Sustainability, Sustainability-oriented innovation, Bio-based production, synthetic biology |
| 201 | [(Marvik and Philp, 2020)](https://paperpile.com/c/L9hHvg/pwEh) | The systemic challenge of the bioeconomy |  |  | Bioeconomy, systemic challenge, policy, innovation, food security, energy security, climate resilience, politics, political economy, transition, industrial manufacturing, agriculture, forestry, marine resources, waste management, renewable carbon strategy, land usage, industry policy, taxation, direct market intervention, mandated production, biofuels, value chain policies, gas fermentation, hydrogen production, technology risk, public funding, scale-up facilities, OECD, Norway |
| 202 | [(Wang, Zang and Zhou, 2022)](https://paperpile.com/c/L9hHvg/1fxu) | Synthetic biology: A powerful booster for future agriculture |  |  | Synthetic biology, Agriculture, Breeding, Carbon/nitrogen fixation, microorganisms |
| 203 | [(Sargent *et al.*, 2022)](https://paperpile.com/c/L9hHvg/3Dao) | Synthetic biology and opportunities within agricultural crops |  |  | Synthetic biology, agricultural crops, crop yields, genetic engineering, crop productivity, fungal diseases, insect pests, heat stress, drought stress, nutrient acquisition, SynBio toolkit, CRISPR-Cas9, gene editing, Golden Gate, gene assembly, RNAi, gene silencing, gene drives, gene synthesis, regulated promoters, artificial promoters, biopesticides, photosynthetic carbon assimilation, Water use efficiency (WUE), photosynthetic enhancement, introducing enzymes, enhancing existing microbial activity, Establishing rhizobium–legume-like interactions |
| 204 | [(Sheahan and Wieden, 2021)](https://paperpile.com/c/L9hHvg/MJ5B) | Emerging regulatory challenges of next-generation synthetic biology |  |  | biologie de synthèse de nouvelle génération; biotechnology policy and regulation; cell-free systems; life-similar systems; next-generation synthetic biology; politique et réglementation en matière de biotechnologie; systèmes acellulaires; systèmes similaires au vivant |

# References

[Achim, R. and Zhang, X. (2022) ‘Exploring the social, ethical, legal, and responsibility dimensions of artificial intelligence for health-a new column in Intelligent Medicine’, *Intelligent Medicine* [Preprint]. Available at:](http://paperpile.com/b/L9hHvg/nY2v) <https://mednexus.org/doi/10.1016/j.imed.2021.12.002> [(Accessed: 27 November 2023).](http://paperpile.com/b/L9hHvg/nY2v)

[A Dixon, T., C Curach, N. and Pretorius, I.S. (2020) ‘Bio-informational futures: The convergence of artificial intelligence and synthetic biology’, *EMBO reports*, 21(3), p. e50036.](http://paperpile.com/b/L9hHvg/D1u4)

[Ahmed, F. and Harrison, C. (2021) ‘Challenges and competencies of entrepreneurial leaders in driving innovation at DIY laboratories’, *Technology Analysis & Strategic Management*, 33(10), pp. 1132–1146.](http://paperpile.com/b/L9hHvg/sg7y)

[Alexander Hamilton, R. *et al.* (2021) *Opportunities, Challenges, and Future Considerations for Top-Down Governance for Biosecurity and Synthetic Biology*. Springer.](http://paperpile.com/b/L9hHvg/a9k6)

[Aparicio, A. (2021) ‘“That would break the containment”: the co-production of responsibility and safety-by-design in xenobiology’, *Journal of Responsible Innovation*, 8(1), pp. 6–27.](http://paperpile.com/b/L9hHvg/Bwqw)

[Asin-Garcia, E. *et al.* (2020) ‘Genetic Safeguards for Safety-by-design: So Close Yet So Far’, *Trends in biotechnology*, 38(12), pp. 1308–1312.](http://paperpile.com/b/L9hHvg/qhZ3)

[Ataii, N. *et al.* (2023) ‘Enabling AI in Synthetic Biology through Construction File Specification’, *bioRxiv*. Available at: https://doi.org/](http://paperpile.com/b/L9hHvg/jADd)[10.1101/2023.06.28.546630](http://dx.doi.org/10.1101/2023.06.28.546630)[.](http://paperpile.com/b/L9hHvg/jADd)

[Baker, B. (2017) ‘Synthetic Biology and the Marketplace: Building the new bioeconomy’, *Bioscience*, 67(10), pp. 877–883.](http://paperpile.com/b/L9hHvg/qJEU)

[Banda, G. and Huzair, F. (2021) ‘Introduction to special issue: innovation/governance interactions in the bioeconomy’, *Technology Analysis & Strategic Management*, 33(3), pp. 257–259.](http://paperpile.com/b/L9hHvg/j7I2)

[Bauer, A. and Bogner, A. (2020) ‘Let’s (not) talk about synthetic biology: Framing an emerging technology in public and stakeholder dialogues’, *Public understanding of science* , 29(5), pp. 492–507.](http://paperpile.com/b/L9hHvg/0bV4)

[Beardall, W.A.V., Stan, G.-B. and Dunlop, M.J. (2022) ‘Deep Learning Concepts and Applications for Synthetic Biology’, *GEN biotechnology*, 1(4), pp. 360–371.](http://paperpile.com/b/L9hHvg/S36y)

[Beeckman, D.S.A. and Rüdelsheim, P. (2020) ‘Biosafety and Biosecurity in Containment: A Regulatory Overview’, *Frontiers in bioengineering and biotechnology*, 8, p. 650.](http://paperpile.com/b/L9hHvg/2zNU)

[Belda, I. *et al.* (2021) ‘Seeding the idea of encapsulating a representative synthetic metagenome in a single yeast cell’, *Nature communications*, 12(1), p. 1599.](http://paperpile.com/b/L9hHvg/khRJ)

[Bhardwaj, A., Kishore, S. and Pandey, D.K. (2022) ‘Artificial Intelligence in Biological Sciences’, *Life* , 12(9). Available at: https://doi.org/](http://paperpile.com/b/L9hHvg/5FxZ)[10.3390/life12091430](http://dx.doi.org/10.3390/life12091430)[.](http://paperpile.com/b/L9hHvg/5FxZ)

[Bianchini, F. (2023) ‘A New Definition of “Artificial” for Two Artificial Sciences’, *Foundations of science*, 28(1), pp. 401–417.](http://paperpile.com/b/L9hHvg/J3LP)

[Blackiston, D. *et al.* (2023) ‘Biological Robots: Perspectives on an Emerging Interdisciplinary Field’, *Soft robotics*, 10(4), pp. 674–686.](http://paperpile.com/b/L9hHvg/AON5)

[Blok, V. and von Schomberg, L. (2023) ‘Introduction’, in V. Blok (ed.) *Putting Responsible Research and Innovation into Practice: A Multi-Stakeholder Approach*. Cham: Springer International Publishing, pp. 1–7.](http://paperpile.com/b/L9hHvg/lewG)

[Bohua, L. *et al.* (2023) ‘Ethical framework on risk governance of synthetic biology’, *Journal of Biosafety and Biosecurity*, 5(2), pp. 45–56.](http://paperpile.com/b/L9hHvg/JOgZ)

[Bongard, J. and Levin, M. (2023) ‘There’s Plenty of Room Right Here: Biological Systems as Evolved, Overloaded, Multi-Scale Machines’, *Biomimetics*, 8(1). Available at: https://doi.org/](http://paperpile.com/b/L9hHvg/uLoS)[10.3390/biomimetics8010110](http://dx.doi.org/10.3390/biomimetics8010110)[.](http://paperpile.com/b/L9hHvg/uLoS)

[Boyd, M. and Wilson, N. (2020) ‘Existential Risks to Humanity Should Concern International Policymakers and More Could Be Done in Considering Them at the International Governance Level’, *Risk analysis: an official publication of the Society for Risk Analysis*, 40(11), pp. 2303–2312.](http://paperpile.com/b/L9hHvg/R43V)

[Bray, D. (2023) *Artificial Intelligence and Synthetic Biology Are Not Harbingers of Doom*. Stimson. Available at:](http://paperpile.com/b/L9hHvg/Sihx) <https://www.stimson.org/2023/artificial-intelligence-and-synthetic-biology-are-not-harbingers-of-doom/> [(Accessed: 27 November 2023).](http://paperpile.com/b/L9hHvg/Sihx)

[Bröring, S., Laibach, N. and Wustmans, M. (2020) ‘Innovation types in the bioeconomy’, *Journal of cleaner production*, 266, p. 121939.](http://paperpile.com/b/L9hHvg/y7g3)

[Burgos-Morales, O. *et al.* (2021) ‘Synthetic biology as driver for the biologization of materials sciences’, *Materials today. Bio*, 11, p. 100115.](http://paperpile.com/b/L9hHvg/c9x1)

[Camacho, D.M. *et al.* (2018) ‘Next-Generation Machine Learning for Biological Networks’, *Cell*, 173(7), pp. 1581–1592.](http://paperpile.com/b/L9hHvg/odAg)

[Capponi, S. and Daniels, K.G. (2023) ‘Harnessing the power of artificial intelligence to advance cell therapy’, *Immunological reviews*, 320(1), pp. 147–165.](http://paperpile.com/b/L9hHvg/9A8g)

[Carbonell, P., Radivojevic, T. and García Martín, H. (2019) ‘Opportunities at the Intersection of Synthetic Biology, Machine Learning, and Automation’, *ACS synthetic biology*, 8(7), pp. 1474–1477.](http://paperpile.com/b/L9hHvg/N20E)

[Carter, S.R. *et al.* (2023) *The Convergence of Artificial Intelligence and the Life Sciences*, *The Nuclear Threat Initiative*. NTI. Available at:](http://paperpile.com/b/L9hHvg/yzyO) <https://www.nti.org/analysis/articles/the-convergence-of-artificial-intelligence-and-the-life-sciences/> [(Accessed: 27 November 2023).](http://paperpile.com/b/L9hHvg/yzyO)

[Cho, M.K. and Relman, D.A. (2010) ‘Genetic technologies. Synthetic “life,” ethics, national security, and public discourse’, *Science*, pp. 38–39.](http://paperpile.com/b/L9hHvg/tdIt)

[Clusmann, J. *et al.* (2023) ‘The future landscape of large language models in medicine’, *Communication & medicine*, 3(1), p. 141.](http://paperpile.com/b/L9hHvg/WSqD)

[Cubillos-Ruiz, A. *et al.* (2021) ‘Engineering living therapeutics with synthetic biology’, *Nature reviews. Drug discovery*, 20(12), pp. 941–960.](http://paperpile.com/b/L9hHvg/L3qc)

[D’Alessandro, W., Lloyd, H.R. and Sharadin, N. (2023) ‘Large Language Models and Biorisk’, *The American journal of bioethics: AJOB*, 23(10), pp. 115–118.](http://paperpile.com/b/L9hHvg/Sz0r)

[Damiano, L., Kuruma, Y. and Stano, P. (2016) ‘What can synthetic biology offer to artificial intelligence (and vice versa)?’, *Bio Systems*, 148, pp. 1–3.](http://paperpile.com/b/L9hHvg/7nOu)

[Damiano, L. and Stano, P. (2023) ‘Explorative Synthetic Biology in AI: Criteria of Relevance and a Taxonomy for Synthetic Models of Living and Cognitive Processes’, *Artificial life*, 29(3), pp. 367–387.](http://paperpile.com/b/L9hHvg/JME3)

[Damoah, I.S. and Botchie, D. (2021) ‘Do-It-Yourself (DIY) laboratories and science, technology, and innovation (STI): trends, implications and future research’, *Technology Analysis & Strategic Management*, 33(10), pp. 1267–1280.](http://paperpile.com/b/L9hHvg/GRWG)

[David, F. *et al.* (2021) ‘A Perspective on Synthetic Biology in Drug Discovery and Development-Current Impact and Future Opportunities’, *SLAS discovery : advancing life sciences R & D*, 26(5), pp. 581–603.](http://paperpile.com/b/L9hHvg/uOZk)

[DiEuliis, D. *et al.* (2019) ‘Does Biotechnology Pose New Catastrophic Risks?’, *Current topics in microbiology and immunology*, 424, pp. 107–119.](http://paperpile.com/b/L9hHvg/uxEg)

[Dixon, T.A. *et al.* (2022) ‘A global forum on synthetic biology: the need for international engagement’, *Nature communications*, 13(1), p. 3516.](http://paperpile.com/b/L9hHvg/5qcR)

[Dixon, T.A. (2023) ‘The bioinformational dilemma: where bioinformational diplomacy meets cyberbiosecurity’, *Australian Journal of International Affairs*, 77(2), pp. 169–187.](http://paperpile.com/b/L9hHvg/ZXEY)

[Dixon, T.A. and Pretorius, I.S. (2020) ‘Drawing on the Past to Shape the Future of Synthetic Yeast Research’, *International journal of molecular sciences*, 21(19). Available at: https://doi.org/](http://paperpile.com/b/L9hHvg/dCcH)[10.3390/ijms21197156](http://dx.doi.org/10.3390/ijms21197156)[.](http://paperpile.com/b/L9hHvg/dCcH)

[Dixon, T.A., Walker, R.S.K. and Pretorius, I.S. (2023) ‘Visioning synthetic futures for yeast research within the context of current global techno-political trends’, *Yeast* , 40(10), pp. 443–456.](http://paperpile.com/b/L9hHvg/BaC7)

[Djeffal, C., Siewert, M.B. and Wurster, S. (2022) ‘Role of the state and responsibility in governing artificial intelligence: a comparative analysis of AI strategies’, *Journal of European Public Policy*, 29(11), pp. 1799–1821.](http://paperpile.com/b/L9hHvg/yyaae)

[van Doren, D. *et al.* (2022) ‘The external commercialisation of technology in emerging domains – the antecedents, consequences, and dimensions of desorptive capacity’, *Technology Analysis & Strategic Management*, 34(3), pp. 258–273.](http://paperpile.com/b/L9hHvg/uadh)

[Dzandu, M.D. and Pathak, B. (2021) ‘Diy laboratories, their practices, and challenges – a systematic literature review’, *Technology Analysis & Strategic Management*, 33(10), pp. 1242–1254.](http://paperpile.com/b/L9hHvg/U6LM)

[Ebrahimkhani, M.R. and Levin, M. (2021) ‘Synthetic living machines: A new window on life’, *iScience*, 24(5), p. 102505.](http://paperpile.com/b/L9hHvg/i3wh)

[Edwards, B. (2014) ‘Taking stock of security concerns related to synthetic biology in an age of responsible innovation’, *Frontiers in public health*, 2, p. 79.](http://paperpile.com/b/L9hHvg/rHKu)

[El Karoui, M., Hoyos-Flight, M. and Fletcher, L. (2019) ‘Future Trends in Synthetic Biology-A Report’, *Frontiers in bioengineering and biotechnology*, 7, p. 175.](http://paperpile.com/b/L9hHvg/sVRY)

[Endy, D. (2005) ‘Foundations for engineering biology’, *Nature*, 438(7067), pp. 449–453.](http://paperpile.com/b/L9hHvg/Ul6q)

[Eslami, M. *et al.* (2022) ‘Artificial intelligence for synthetic biology’, *Communications of the ACM*, 65(5), pp. 88–97.](http://paperpile.com/b/L9hHvg/HxPt)

[Esquivel-Sada, D. (2022) ‘Responsible intellectual property rights? Untangling open-source biotech adherence to intellectual property rights through DIYbio’, *Technology in society*, 70, p. 102005.](http://paperpile.com/b/L9hHvg/ihM5)

[Evans, S.W. (, 2022) ‘When All Research Is Dual Use’, *Issues in science and technology*, 38(3). Available at:](http://paperpile.com/b/L9hHvg/3Caj) <https://issues.org/dual-use-research-biosecurity-social-context-science-evans/> [(Accessed: 28 November 2023).](http://paperpile.com/b/L9hHvg/3Caj)

[Fantine, A.L., Margis, R., Talamini, E.l and Dewes, H. (2023) ‘Trends in Synthetic Biology in the Bioeconomy of Non-Food-Competing Biofuels’, *SynBio*, 1(1). Available at:](http://paperpile.com/b/L9hHvg/e0z2) <https://www.mdpi.com/2674-0583/1/1/3>[.](http://paperpile.com/b/L9hHvg/e0z2)

[Freemont, P.S. (2019) ‘Synthetic biology industry: data-driven design is creating new opportunities in biotechnology’, *Emerging topics in life sciences*, 3(5), pp. 651–657.](http://paperpile.com/b/L9hHvg/6f9q)

[Frow, E. (2020) ‘From “Experiments of Concern” to “Groups of Concern”: Constructing and Containing Citizens in Synthetic Biology’, *Science, technology & human values*, 45(6), pp. 1038–1064.](http://paperpile.com/b/L9hHvg/hICl)

[Fulvi, D. and Wodak, J. (2023) ‘Gambling on unknown unknowns: Risk ethics for a climate change technofix’, *The Anthropocene Review*, p. 20530196231204324.](http://paperpile.com/b/L9hHvg/XiIA)

[Garner, K.L. (2021) ‘Principles of synthetic biology’, *Essays in biochemistry*, 65(5), pp. 791–811.](http://paperpile.com/b/L9hHvg/nrRC)

[‘Generating “smarter” biotechnology’ (2023) *Nature biotechnology*, 41(2), p. 157.](http://paperpile.com/b/L9hHvg/zNOH)

[Getz, L.J. and Dellaire, G. (2018) ‘Angels and Devils: Dilemmas in Dual-Use Biotechnology’, *Trends in biotechnology*, 36(12), pp. 1202–1205.](http://paperpile.com/b/L9hHvg/Uulp)

[Gisselsson, D. (2022) ‘Next-Generation Biowarfare: Small in Scale, Sensational in Nature?’, *Health security*, 20(2), pp. 182–186.](http://paperpile.com/b/L9hHvg/9zlZ)

[Gomez-Hinostroza, E.S. *et al.* (2023) ‘Current landscape and future directions of synthetic biology in South America’, *Frontiers in bioengineering and biotechnology*, 11, p. 1069628.](http://paperpile.com/b/L9hHvg/0EmX)

[Grinbaum, A. and Adomaitis, L. (2023) ‘Dual Use Concerns of Generative AI and Large Language Models’, *arXiv [cs.CY]*. Available at:](http://paperpile.com/b/L9hHvg/A148) <http://arxiv.org/abs/2305.07882>[.](http://paperpile.com/b/L9hHvg/A148)

[Gronvall, G.K. (2018) ‘Safety, security, and serving the public interest in synthetic biology’, *Journal of industrial microbiology & biotechnology*, 45(7), pp. 463–466.](http://paperpile.com/b/L9hHvg/OQc3)

[Hagendorff, T. (2021) ‘Forbidden knowledge in machine learning reflections on the limits of research and publication’, *AI & society*, 36(3), pp. 767–781.](http://paperpile.com/b/L9hHvg/e7j0)

[Hamlyn, O. (2022) ‘Synthesize this: integrating innovation governance and EU regulation of synthetic biology’, *Journal of law and society*, 49(3), pp. 577–602.](http://paperpile.com/b/L9hHvg/TsCT)

[Hammang, A. (2023) ‘Troubleshooting: The Automation of Synthetic Biology and the Labor of Technological Futures’, *Science, technology & human values*, p. 01622439221149524.](http://paperpile.com/b/L9hHvg/mx9D)

[Hanczyc, M.M. (2020) ‘Engineering Life: A Review of Synthetic Biology’, *Artificial life*, 26(2), pp. 260–273.](http://paperpile.com/b/L9hHvg/iHje)

[Hanson, A.D. and Lorenzo, V. de (2023) ‘Synthetic Biology─High Time to Deliver?’, *ACS synthetic biology*, 12(6), pp. 1579–1582.](http://paperpile.com/b/L9hHvg/taYX)

[Harrer, S. (2023) ‘Attention is not all you need: the complicated case of ethically using large language models in healthcare and medicine’, *EBioMedicine*, 90, p. 104512.](http://paperpile.com/b/L9hHvg/aQ9u)

[Hassoun, S. *et al.* (2022) ‘Artificial Intelligence for Biology’, *Integrative and comparative biology*, 61(6), pp. 2267–2275.](http://paperpile.com/b/L9hHvg/McGr)

[Helmy, M., Smith, D. and Selvarajoo, K. (2020) ‘Systems biology approaches integrated with artificial intelligence for optimized metabolic engineering’, *Metabolic engineering communications*, 11, p. e00149.](http://paperpile.com/b/L9hHvg/jL1H)

[Heyndrickx, W. *et al.* (2023) ‘MELLODDY: Cross-pharma Federated Learning at Unprecedented Scale Unlocks Benefits in QSAR without Compromising Proprietary Information’, *Journal of chemical information and modeling* [Preprint]. Available at: https://doi.org/](http://paperpile.com/b/L9hHvg/iOaZ)[10.1021/acs.jcim.3c00799](http://dx.doi.org/10.1021/acs.jcim.3c00799)[.](http://paperpile.com/b/L9hHvg/iOaZ)

[Hillson, N. *et al.* (2019) ‘Building a global alliance of biofoundries’, *Nature communications*, 10(1), p. 2040.](http://paperpile.com/b/L9hHvg/KAAy)

[Hınçer, A. *et al.* (2023) ‘Making the Next Generation of Therapeutics: mRNA Meets Synthetic Biology’, *ACS synthetic biology*, 12(9), pp. 2505–2515.](http://paperpile.com/b/L9hHvg/I7nT)

[Hoffmann, A. (2023) ‘Designer genes courtesy of artificial intelligence’, *Genes & development*, 37(9-10), pp. 351–353.](http://paperpile.com/b/L9hHvg/iCj0)

[Hoffmann, S.A. *et al.* (2023) ‘Safety by design: Biosafety and biosecurity in the age of synthetic genomics’, *iScience*, 26(3), p. 106165.](http://paperpile.com/b/L9hHvg/JGH6)

[Holland, C. *et al.* (2024) ‘Innovation intermediaries at the convergence of digital technologies, sustainability, and governance: A case study of AI-enabled engineering biology’, *Technovation*, 129, p. 102875.](http://paperpile.com/b/L9hHvg/U95f)

[Holzinger, A. *et al.* (2023) ‘AI for life: Trends in artificial intelligence for biotechnology’, *New biotechnology*, 74, pp. 16–24.](http://paperpile.com/b/L9hHvg/SMy4)

[Huzair, F. (2021) ‘Risk and regulatory culture: governing recombinant DNA technology in the UK from 1970–1980’, *Technology Analysis & Strategic Management*, 33(3), pp. 260–270.](http://paperpile.com/b/L9hHvg/cYOd)

[Ienca, M. and Vayena, E. (2018) ‘Dual use in the 21st century: emerging risks and global governance’, *Swiss medical weekly*, 148, p. w14688.](http://paperpile.com/b/L9hHvg/OKor)

[Jafari, M. *et al.* (2023) ‘Merging machine learning and bioelectronics for closed-loop control of biological systems and homeostasis’, *Cell Reports Physical Science*, 4(8), p. 101535.](http://paperpile.com/b/L9hHvg/CQEO)

[Jatain, I. *et al.* (2021) ‘Synthetic biology potential for carbon sequestration into biocommodities’, *Journal of cleaner production*, 323, p. 129176.](http://paperpile.com/b/L9hHvg/OUKs)

[Jin, Q. *et al.* (2023) ‘GeneGPT: Augmenting Large Language Models with Domain Tools for Improved Access to Biomedical Information’, *ArXiv* [Preprint]. Available at:](http://paperpile.com/b/L9hHvg/HkFo) <https://www.ncbi.nlm.nih.gov/pubmed/37131884>[.](http://paperpile.com/b/L9hHvg/HkFo)

[Kahl, L.J. and Endy, D. (2013) ‘A survey of enabling technologies in synthetic biology’, *Journal of biological engineering*, 7(1), p. 13.](http://paperpile.com/b/L9hHvg/HMs1)

[Kather, J.N. *et al.* (2022) ‘Medical domain knowledge in domain-agnostic generative AI’, *NPJ digital medicine*, 5(1), p. 90.](http://paperpile.com/b/L9hHvg/Y6Ck)

[Keiper, F. and Atanassova, A. (2020) ‘Regulation of Synthetic Biology: Developments Under the Convention on Biological Diversity and Its Protocols’, *Frontiers in bioengineering and biotechnology*, 8, p. 310.](http://paperpile.com/b/L9hHvg/aIQP)

[Kirksey, E. (2021) ‘Living Machines Go Wild: Policing the Imaginative Horizons of Synthetic Biology’, *Current anthropology*, 62(S24), pp. S287–S297.](http://paperpile.com/b/L9hHvg/sezo)

[Kitano, S. *et al.* (2023) ‘Synthetic biology: Learning the way toward high-precision biological design’, *PLoS biology*, 21(4), p. e3002116.](http://paperpile.com/b/L9hHvg/9um6)

[Koskinen, R. (2020) ‘Could We Really Be Made of Swiss Cheese? Xenobiology as an Engineering Epistemology for Biological Realization’, *Chembiochem: a European journal of chemical biology*, 21(18), pp. 2591–2594.](http://paperpile.com/b/L9hHvg/5QHt)

[Kumar, P., Sinha, R. and Shukla, P. (2022) ‘Artificial intelligence and synthetic biology approaches for human gut microbiome’, *Critical reviews in food science and nutrition*, 62(8), pp. 2103–2121.](http://paperpile.com/b/L9hHvg/NKUg)

[Lawrence, D.R. (2019) ‘Advanced bioscience and AI: debugging the future of life’, *Emerging topics in life sciences*, 3(6), pp. 747–751.](http://paperpile.com/b/L9hHvg/DVLE)

[Lawson, C.E. *et al.* (2021) ‘Machine learning for metabolic engineering: A review’, *Metabolic engineering*, 63, pp. 34–60.](http://paperpile.com/b/L9hHvg/ApSV)

[Lee, S.J. and Kim, D.-M. (2023) ‘Cell-Free Synthetic Biology: Navigating the New Frontiers of Biomanufacturing and Biological Engineering’, *Current Opinion in Systems Biology*, p. 100488.](http://paperpile.com/b/L9hHvg/RstJ)

[Lee, T. and George, J. (2023) ‘Future worldbuilding with synthetic biology: A case study in interdisciplinary scenario visualization’, *Futures*, 147, p. 103118.](http://paperpile.com/b/L9hHvg/CaP1)

[Lev, O. (2019) ‘Regulating dual-use research: Lessons from Israel and the United States’, *Journal of Biosafety and Biosecurity*, 1(2), pp. 80–85.](http://paperpile.com/b/L9hHvg/tZwu)

[Liedong, T.A. and Sarpong, D. (2021) ‘Taking stock and charting the future: the management and implications of DIY laboratories for innovation and society’, *Technology Analysis & Strategic Management*, 33(10), pp. 1119–1131.](http://paperpile.com/b/L9hHvg/gEda)

[Li, J. *et al.* (2021) ‘Advances in Synthetic Biology and Biosafety Governance’, *Frontiers in bioengineering and biotechnology*, 9, p. 598087.](http://paperpile.com/b/L9hHvg/Gq8K)

[Li, J. *et al.* (2023) ‘ChatGPT in healthcare: A taxonomy and systematic review’, *bioRxiv*. Available at: https://doi.org/](http://paperpile.com/b/L9hHvg/jsU6)[10.1101/2023.03.30.23287899](http://dx.doi.org/10.1101/2023.03.30.23287899)[.](http://paperpile.com/b/L9hHvg/jsU6)

[Linkov, I., Trump, B.D., Anklam, E., *et al.* (2018) ‘Comparative, collaborative, and integrative risk governance for emerging technologies’, *Environment systems & decisions*, 38(2), pp. 170–176.](http://paperpile.com/b/L9hHvg/ohqD)

[Linkov, I., Trump, B.D., Poinsatte-Jones, K., *et al.* (2018) ‘Governance Strategies for a Sustainable Digital World’, *Sustainability: Science Practice and Policy*, 10(2), p. 440.](http://paperpile.com/b/L9hHvg/9YBI)

[Li, Z. *et al.* (2022) ‘An Account of Models of Molecular Circuits for Associative Learning with Reinforcement Effect and Forced Dissociation’, *Sensors* , 22(15). Available at: https://doi.org/](http://paperpile.com/b/L9hHvg/OMXN)[10.3390/s22155907](http://dx.doi.org/10.3390/s22155907)[.](http://paperpile.com/b/L9hHvg/OMXN)

[Lockhart, A., Marvin, S. and While, A. (2023) ‘Towards new ecologies of automation: Robotics and the re-engineering of nature’, *Geoforum; journal of physical, human, and regional geosciences*, 145, p. 103825.](http://paperpile.com/b/L9hHvg/zg0o)

[de Lorenzo, V., Krasnogor, N. and Schmidt, M. (2021) ‘For the sake of the Bioeconomy: define what a Synthetic Biology Chassis is!’, *New biotechnology*, 60, pp. 44–51.](http://paperpile.com/b/L9hHvg/9hjw)

[Lubiana, T. *et al.* (2023) ‘Ten quick tips for harnessing the power of ChatGPT in computational biology’, *PLoS computational biology*, 19(8), p. e1011319.](http://paperpile.com/b/L9hHvg/F2JP)

[Luo, R. *et al.* (2022) ‘BioGPT: generative pre-trained transformer for biomedical text generation and mining’, *Briefings in bioinformatics*, 23(6). Available at: https://doi.org/](http://paperpile.com/b/L9hHvg/30cA)[10.1093/bib/bbac409](http://dx.doi.org/10.1093/bib/bbac409)[.](http://paperpile.com/b/L9hHvg/30cA)

[Mahesh, N. *et al.* (2023) ‘Viable remediation techniques to cleansing wastewaters comprising endocrine-disrupting compounds’, *Environmental research*, 231(Pt 3), p. 116245.](http://paperpile.com/b/L9hHvg/j5SG)

[Mampuys, R. and Brom, F. (2018) ‘Emerging crossover technologies: How to organize a biotechnology that becomes mainstream?’, *Environment Systems and Decisions*, 38(2), pp. 163–169.](http://paperpile.com/b/L9hHvg/qBt2)

[Marris, C. and Calvert, J. (2020) ‘Science and Technology Studies in Policy: The UK Synthetic Biology Roadmap’, *Science, technology & human values*, 45(1), pp. 34–61.](http://paperpile.com/b/L9hHvg/GZlx)

[Martin, H.G. *et al.* (2023) ‘Perspectives for self-driving labs in synthetic biology’, *Current opinion in biotechnology*, 79, p. 102881.](http://paperpile.com/b/L9hHvg/sEli)

[Marvik, O.J. and Philp, J. (2020) ‘The systemic challenge of the bioeconomy: A policy framework for transitioning towards a sustainable carbon cycle economy’, *EMBO reports*, 21(10), p. e51478.](http://paperpile.com/b/L9hHvg/pwEh)

[Mateu-Sanz, M. *et al.* (2023) ‘Redefining biomaterial biocompatibility: challenges for artificial intelligence and text mining’, *Trends in biotechnology* [Preprint]. Available at: https://doi.org/](http://paperpile.com/b/L9hHvg/nILc)[10.1016/j.tibtech.2023.09.015](http://dx.doi.org/10.1016/j.tibtech.2023.09.015)[.](http://paperpile.com/b/L9hHvg/nILc)

[Matsuyama, H. and Suzuki, H.I. (2019) ‘Systems and Synthetic microRNA Biology: From Biogenesis to Disease Pathogenesis’, *International journal of molecular sciences*, 21(1). Available at: https://doi.org/](http://paperpile.com/b/L9hHvg/8KTT)[10.3390/ijms21010132](http://dx.doi.org/10.3390/ijms21010132)[.](http://paperpile.com/b/L9hHvg/8KTT)

[Meissner, D. *et al.* (2021) ‘The rise of do-it-yourself (DiY) laboratories: Implications for science, technology, and innovation (STI) policy’, *Technological forecasting and social change*, 165, p. 120589.](http://paperpile.com/b/L9hHvg/Kdmd)

[Merchant, A. *et al.* (2023) ‘Scaling deep learning for materials discovery’, *Nature* [Preprint]. Available at: https://doi.org/](http://paperpile.com/b/L9hHvg/bRUB)[10.1038/s41586-023-06735-9](http://dx.doi.org/10.1038/s41586-023-06735-9)[.](http://paperpile.com/b/L9hHvg/bRUB)

[Mervin, L.H. *et al.* (2021) ‘Uncertainty quantification in drug design’, *Drug discovery today*, 26(2), pp. 474–489.](http://paperpile.com/b/L9hHvg/BCrh)

[Mialon, G. *et al.* (2023) ‘GAIA: a benchmark for General AI Assistants’, *arXiv [cs.CL]*. Available at:](http://paperpile.com/b/L9hHvg/IUsY) <http://arxiv.org/abs/2311.12983>[.](http://paperpile.com/b/L9hHvg/IUsY)

[Millett, P. *et al.* (2020) ‘The synthetic-biology challenges for biosecurity: examples from iGEM’, *The Nonproliferation Review*, 27(4-6), pp. 443–458.](http://paperpile.com/b/L9hHvg/5nUb)

[Millett, P. and Alexanian, T. (2021) ‘Implementing adaptive risk management for synthetic biology: Lessons from iGEM’s safety and security programme’, *Engineering biology*, 5(3), pp. 64–71.](http://paperpile.com/b/L9hHvg/1A46)

[Mökander, J. *et al.* (2022) ‘Challenges and best practices in corporate AI governance: Lessons from the biopharmaceutical industry’, *Frontiers in Computer Science*, 4. Available at: https://doi.org/](http://paperpile.com/b/L9hHvg/Z9FJ)[10.3389/fcomp.2022.1068361](http://dx.doi.org/10.3389/fcomp.2022.1068361)[.](http://paperpile.com/b/L9hHvg/Z9FJ)

[Moon, T.S. (2023) ‘EBRC: Enhancing bioeconomy through research and communication’, *New biotechnology*, 78, pp. 150–152.](http://paperpile.com/b/L9hHvg/g3IO)

[Morris, M.R. (2023) ‘Scientists’ Perspectives on the Potential for Generative AI in their Fields’, *arXiv [cs.CY]*. Available at:](http://paperpile.com/b/L9hHvg/uUxR) <http://arxiv.org/abs/2304.01420>[.](http://paperpile.com/b/L9hHvg/uUxR)

[Mourby, M. *et al.* (2022) ‘Biomodifying the “natural”: from Adaptive Regulation to Adaptive Societal Governance’, *Journal of law and the biosciences*, 9(1), p. lsac018.](http://paperpile.com/b/L9hHvg/RbNN)

[Murashov, V., Howard, J. and Schulte, P. (2020) ‘Synthetic Biology Industry: Biosafety Risks to Workers’, in B.D. Trump et al. (eds) *Synthetic Biology 2020: Frontiers in Risk Analysis and Governance*. Cham: Springer International Publishing, pp. 165–182.](http://paperpile.com/b/L9hHvg/yQG7)

[Naderi Yeganeh, P. *et al.* (2023) ‘PanomiR: a systems biology framework for analysis of multi-pathway targeting by miRNAs’, *Briefings in bioinformatics*, 24(6). Available at: https://doi.org/](http://paperpile.com/b/L9hHvg/bxtm)[10.1093/bib/bbad418](http://dx.doi.org/10.1093/bib/bbad418)[.](http://paperpile.com/b/L9hHvg/bxtm)

[Nelson, C. *et al.* (2021) ‘Foresight in Synthetic Biology and Biotechnology Threats’, in B.D. Trump et al. (eds) *Emerging Threats of Synthetic Biology and Biotechnology: Addressing Security and Resilience Issues*. Dordrecht (DE): Springer.](http://paperpile.com/b/L9hHvg/vA0S)

[Nesbeth, D.N. *et al.* (2016) ‘Synthetic biology routes to bio-artificial intelligence’, *Essays in biochemistry*, 60(4), pp. 381–391.](http://paperpile.com/b/L9hHvg/w4LE)

[Nguyen, D.-H. *et al.* (2023) ‘Bioengineering of bacteria for cancer immunotherapy’, *Nature communications*, 14(1), p. 3553.](http://paperpile.com/b/L9hHvg/GkhL)

[Nylund, P.A. *et al.* (2022) ‘The emergence of entrepreneurial ecosystems based on enabling technologies: Evidence from synthetic biology’, *Journal of business research*, 149, pp. 728–735.](http://paperpile.com/b/L9hHvg/1ayF)

[O’Brien, J.T. and Nelson, C. (2020) ‘Assessing the Risks Posed by the Convergence of Artificial Intelligence and Biotechnology’, *Health security*, 18(3), pp. 219–227.](http://paperpile.com/b/L9hHvg/aNfE)

[Oliveira, S.M.D. and Densmore, D. (2022) ‘Hardware, Software, and Wetware Codesign Environment for Synthetic Biology’, *Biodesign research*, 2022, p. 9794510.](http://paperpile.com/b/L9hHvg/4HYA)

[Ou, Y. and Guo, S. (2023) ‘Safety risks and ethical governance of biomedical applications of synthetic biology’, *Frontiers in bioengineering and biotechnology*, 11, p. 1292029.](http://paperpile.com/b/L9hHvg/PZPB)

[Palmer, M.J., Fukuyama, F. and Relman, D.A. (2015) ‘SCIENCE GOVERNANCE. A more systematic approach to biological risk’, *Science*, 350(6267), pp. 1471–1473.](http://paperpile.com/b/L9hHvg/isuj)

[Pannu, J. *et al.* (2022) ‘Strengthen oversight of risky research on pathogens’, *Science*, 378(6625), pp. 1170–1172.](http://paperpile.com/b/L9hHvg/PpYg)

[Pansera, M. *et al.* (2020) ‘Embedding responsible innovation within synthetic biology research and innovation: insights from a UK multi-disciplinary research centre’, *Journal of Responsible Innovation*, 7(3), pp. 384–409.](http://paperpile.com/b/L9hHvg/DcZn)

[Patel, V.L. *et al.* (2009) ‘The coming of age of artificial intelligence in medicine’, *Artificial intelligence in medicine*, 46(1), pp. 5–17.](http://paperpile.com/b/L9hHvg/UC1d)

[de Pedraza, P. and Vollbracht, I. (2023) ‘General theory of data, artificial intelligence and governance’, *Humanities and Social Sciences Communications*, 10(1), pp. 1–16.](http://paperpile.com/b/L9hHvg/oLU8P)

[Pei, L., Garfinkel, M. and Schmidt, M. (2022) ‘Bottlenecks and opportunities for synthetic biology biosafety standards’, *Nature communications*, 13(1), p. 2175.](http://paperpile.com/b/L9hHvg/xqMy)

[Perrakis, A. and Sixma, T.K. (2021) ‘AI revolutions in biology: The joys and perils of AlphaFold’, *EMBO reports*, 22(11), p. e54046.](http://paperpile.com/b/L9hHvg/DRhp)

[Pio-Lopez, L. (2021) ‘The rise of the biocyborg: synthetic biology, artificial chimerism and human enhancement’, *New genetics and society*, 40(4), pp. 599–619.](http://paperpile.com/b/L9hHvg/UR9c)

[Qiu, Y. and Wei, G.-W. (2023) ‘Artificial intelligence-aided protein engineering: from topological data analysis to deep protein language models’, *Briefings in bioinformatics*, 24(5). Available at: https://doi.org/](http://paperpile.com/b/L9hHvg/XDam)[10.1093/bib/bbad289](http://dx.doi.org/10.1093/bib/bbad289)[.](http://paperpile.com/b/L9hHvg/XDam)

[Qureshi, R. *et al.* (2023) ‘AI in drug discovery and its clinical relevance’, *Heliyon*, 9(7), p. e17575.](http://paperpile.com/b/L9hHvg/1ciR)

[Radivojević, T. *et al.* (2020) ‘A machine learning Automated Recommendation Tool for synthetic biology’, *Nature communications*, 11(1), p. 4879.](http://paperpile.com/b/L9hHvg/tV0j)

[Ray, P.P. (2023) ‘ChatGPT: A comprehensive review on background, applications, key challenges, bias, ethics, limitations and future scope’, *Internet of Things and Cyber-Physical Systems*, 3, pp. 121–154.](http://paperpile.com/b/L9hHvg/ZzuS)

[Rennings, M., Burgsmüller, A.P.F. and Bröring, S. (2023) ‘Convergence towards a digitalized bioeconomy—Exploring cross‐industry merger and acquisition activities between the bioeconomy and the digital economy’, *Business strategy & development*, 6(1), pp. 53–74.](http://paperpile.com/b/L9hHvg/u2Qe)

[Reynolds, J.L. (2021) ‘Engineering biological diversity: the international governance of synthetic biology, gene drives, and de-extinction for conservation’, *Current Opinion in Environmental Sustainability*, 49, pp. 1–6.](http://paperpile.com/b/L9hHvg/zi39)

[Ribeiro, B. *et al.* (2023) ‘The digitalisation paradox of everyday scientific labour: How mundane knowledge work is amplified and diversified in the biosciences’, *Research policy*, 52(1), p. 104607.](http://paperpile.com/b/L9hHvg/poKL)

[Rudolph, A. *et al.* (2023) ‘Strategies to identify and edit improvements in synthetic genome segments episomally’, *Nucleic acids research*, 51(18), pp. 10094–10106.](http://paperpile.com/b/L9hHvg/C4LS)

[Sandbrink, J. (2023) *Panoptic dual-use management: preventing deliberate pandemics in an age of synthetic biology and artificial intelligence*. University of Oxford. Available at: https://doi.org/](http://paperpile.com/b/L9hHvg/lV6s)[10.5287/ORA-BPQK58EAD](http://dx.doi.org/10.5287/ORA-BPQK58EAD)[.](http://paperpile.com/b/L9hHvg/lV6s)

[Sandbrink, J.B. (2023) ‘Artificial intelligence and biological misuse: Differentiating risks of language models and biological design tools’, *arXiv [cs.CY]*. Available at:](http://paperpile.com/b/L9hHvg/S8fi) <http://arxiv.org/abs/2306.13952>[.](http://paperpile.com/b/L9hHvg/S8fi)

[Sargent, D. *et al.* (2022) ‘Synthetic biology and opportunities within agricultural crops’, *Journal of Sustainable Agriculture and Environment*, 1(2), pp. 89–107.](http://paperpile.com/b/L9hHvg/3Dao)

[Sarkar, C. *et al.* (2023) ‘Artificial Intelligence and Machine Learning Technology Driven Modern Drug Discovery and Development’, *International journal of molecular sciences*, 24(3). Available at: https://doi.org/](http://paperpile.com/b/L9hHvg/yoYo)[10.3390/ijms24032026](http://dx.doi.org/10.3390/ijms24032026)[.](http://paperpile.com/b/L9hHvg/yoYo)

[Schaefer, M. *et al.* (2023) ‘Large language models are universal biomedical simulators’, *bioRxiv*. Available at: https://doi.org/](http://paperpile.com/b/L9hHvg/qLY0)[10.1101/2023.06.16.545235](http://dx.doi.org/10.1101/2023.06.16.545235)[.](http://paperpile.com/b/L9hHvg/qLY0)

[Scheper, T. *et al.* (2021) ‘Digitalization and Bioprocessing: Promises and Challenges’, in C. Herwig, R. Pörtner, and J. Möller (eds) *Digital Twins: Tools and Concepts for Smart Biomanufacturing*. Cham: Springer International Publishing, pp. 57–69.](http://paperpile.com/b/L9hHvg/VdDv)

[Schmidt, M. and de Lorenzo, V. (2016) ‘Synthetic bugs on the loose: containment options for deeply engineered (micro)organisms’, *Current opinion in biotechnology*, 38, pp. 90–96.](http://paperpile.com/b/L9hHvg/rV5C)

[Seydel, C. (2023) ‘DNA writing technologies moving toward synthetic genomes’, *Nature biotechnology*, 41(11), pp. 1504–1509.](http://paperpile.com/b/L9hHvg/Mvoe)

[Shapira, P., Kwon, S. and Youtie, J. (2017) ‘Tracking the emergence of synthetic biology’, *Scientometrics*, 112(3), pp. 1439–1469.](http://paperpile.com/b/L9hHvg/HQpy)

[Sheahan, T. and Wieden, H.-J. (2021) ‘Emerging regulatory challenges of next-generation synthetic biology’, *Biochemistry and cell biology = Biochimie et biologie cellulaire*, 99(6), pp. 766–771.](http://paperpile.com/b/L9hHvg/MJ5B)

[Shi, Z. *et al.* (2022) ‘Data-Driven Synthetic Cell Factories Development for Industrial Biomanufacturing’, *Biodesign research*, 2022, p. 9898461.](http://paperpile.com/b/L9hHvg/x62f)

[Soice, E.H. *et al.* (2023) ‘Can large language models democratize access to dual-use biotechnology?’, *arXiv [cs.CY]*. Available at:](http://paperpile.com/b/L9hHvg/TGC8) <http://arxiv.org/abs/2306.03809>[.](http://paperpile.com/b/L9hHvg/TGC8)

[Stano, P. (2023) ‘Chemical Systems for Wetware Artificial Life: Selected Perspectives in Synthetic Cell Research’, *International journal of molecular sciences*, 24(18). Available at: https://doi.org/](http://paperpile.com/b/L9hHvg/jeJ2)[10.3390/ijms241814138](http://dx.doi.org/10.3390/ijms241814138)[.](http://paperpile.com/b/L9hHvg/jeJ2)

[Sundaram, L.S., Ajioka, J.W. and Molloy, J.C. (2023) ‘Synthetic biology regulation in Europe: containment, release and beyond’, *Synthetic Biology*, 8(1), p. ysad009.](http://paperpile.com/b/L9hHvg/yNII)

[Sun, T. *et al.* (2022) ‘Challenges and recent progress in the governance of biosecurity risks in the era of synthetic biology’, *Journal of Biosafety and Biosecurity*, 4(1), pp. 59–67.](http://paperpile.com/b/L9hHvg/zNjW)

[Szocik, K. *et al.* (2021) ‘Future space missions and human enhancement: Medical and ethical challenges’, *Futures*, 133, p. 102819.](http://paperpile.com/b/L9hHvg/UIql)

[Tait, J. and Wield, D. (2021) ‘Policy support for disruptive innovation in the life sciences’, *Technology Analysis & Strategic Management*, 33(3), pp. 307–319.](http://paperpile.com/b/L9hHvg/SG4W)

[Tang, L. *et al.* (2023) ‘Synthetic biology and governance research in China: a 40-year evolution’, *Scientometrics*, 128(9), pp. 5293–5310.](http://paperpile.com/b/L9hHvg/LSBf)

[Tang, T.-C. *et al.* (2020) ‘Materials design by synthetic biology’, *Nature Reviews Materials*, 6(4), pp. 332–350.](http://paperpile.com/b/L9hHvg/anXZ)

[Tarasava, K. (2023) *How AI Is Transforming Synthetic Biology: Reaching Far Beyond Biopharma*. Available at:](http://paperpile.com/b/L9hHvg/1XoG) <https://www.synbiobeta.com/read/how-ai-is-transforming-synthetic-biology-reaching-far-beyond-biopharma> [(Accessed: 28 November 2023).](http://paperpile.com/b/L9hHvg/1XoG)

[Taylor, K. *et al.* (2023) ‘Intrinsic responsible innovation in a synthetic biology research project’, *New genetics and society*, 42(1), p. e2232684.](http://paperpile.com/b/L9hHvg/2xkt)

[Tebeje, A., Tadesse, H. and Mengesha, Y. (2021) ‘Synthetic bio/techno/logy and its application’, *Biotechnology, biotechnological equipment*, 35(1), pp. 1156–1162.](http://paperpile.com/b/L9hHvg/wq0z)

[Thiele, L.P. (2020) ‘Nature 4.0: Assisted evolution, DE-extinction, and ecological restoration technologies’, *Global environmental politics*, 20(3), pp. 9–27.](http://paperpile.com/b/L9hHvg/TA2b)

[Tinafar, A., Jaenes, K. and Pardee, K. (2019) ‘Synthetic Biology Goes Cell-Free’, *BMC biology*, 17(1), p. 64.](http://paperpile.com/b/L9hHvg/iVDm)

[Tong, Y. and Zhang, L. (2023) ‘Discovering the next decade’s synthetic biology research trends with ChatGPT’, *Synthetic and systems biotechnology*, 8(2), pp. 220–223.](http://paperpile.com/b/L9hHvg/Q17l)

[Topol, E.J. (2019) ‘High-performance medicine: the convergence of human and artificial intelligence’, *Nature medicine*, 25(1), pp. 44–56.](http://paperpile.com/b/L9hHvg/sT3j)

[Trump, B. *et al.* (2022) ‘Governing biotechnology to provide safety and security and address ethical, legal, and social implications’, *Frontiers in genetics*, 13, p. 1052371.](http://paperpile.com/b/L9hHvg/vWiK)

[Trump, B.D. *et al.* (2019) ‘Co-evolution of physical and social sciences in synthetic biology’, *Critical reviews in biotechnology*, 39(3), pp. 351–365.](http://paperpile.com/b/L9hHvg/0zpn)

[Trump, B.D. *et al.* (2020) ‘Building biosecurity for synthetic biology’, *Molecular systems biology*, 16(7), p. e9723.](http://paperpile.com/b/L9hHvg/hPvy)

[Urbina, F. *et al.* (2022) ‘Dual Use of Artificial Intelligence-powered Drug Discovery’, *Nature machine intelligence*, 4(3), pp. 189–191.](http://paperpile.com/b/L9hHvg/shnX)

[Vallejos-Romero, A. *et al.* (2021) ‘Social sciences, risks and synthetic biology: A review of the literature’, *Sociología y tecnociencia*, 11(Extra_2), pp. 213–239.](http://paperpile.com/b/L9hHvg/7J7d)

[Vallero Daniel A. and Gunsch Claudia K. (2020) ‘Applications and Implications of Emerging Biotechnologies in Environmental Engineering’, *Journal of Environmental Engineering*, 146(6), p. 03120005.](http://paperpile.com/b/L9hHvg/O1Yp)

[Vaseashta, A. (2023) ‘Existential Risks Associated with Dual-Use Technologies’, in T. Undheim and D. Zimmer (eds) *Proceedings of the Stanford Existential Risks Conference 2023*. Stanford University, pp. 156–170.](http://paperpile.com/b/L9hHvg/Ypyt)

[Vinke, S., Rais, I. and Millett, P. (2022) ‘The Dual-Use Education Gap: Awareness and Education of Life Science Researchers on Nonpathogen-Related Dual-Use Research’, *Health security*, 20(1), pp. 35–42.](http://paperpile.com/b/L9hHvg/eBoD)

[Voigt, C.A. (2020) ‘Synthetic biology 2020-2030: six commercially-available products that are changing our world’, *Nature communications*, 11(1), p. 6379.](http://paperpile.com/b/L9hHvg/YnpI)

[Wang, F. and Zhang, W. (2019) ‘Synthetic biology: Recent progress, biosafety and biosecurity concerns, and possible solutions’, *Journal of Biosafety and Biosecurity*, 1(1), pp. 22–30.](http://paperpile.com/b/L9hHvg/3ylQ)

[Wang, G. *et al.* (2023) ‘Ethical and social insights into synthetic biology: predicting research fronts in the post-COVID-19 era’, *Frontiers in bioengineering and biotechnology*, 11, p. 1085797.](http://paperpile.com/b/L9hHvg/IS5A)

[Wang, L., Zang, X. and Zhou, J. (2022) ‘Synthetic biology: A powerful booster for future agriculture’, *Advanced Agrochem*, 1(1), pp. 7–11.](http://paperpile.com/b/L9hHvg/1fxu)

[Wani, A.K. *et al.* (2022) ‘Metagenomics and artificial intelligence in the context of human health’, *Infection, genetics and evolution: journal of molecular epidemiology and evolutionary genetics in infectious diseases*, 100, p. 105267.](http://paperpile.com/b/L9hHvg/WGsE)

[Watkins, A. *et al.* (2023) ‘Public biofoundries as innovation intermediaries: the integration of translation, sustainability, and responsibility’, *The Journal of technology transfer* [Preprint]. Available at: https://doi.org/](http://paperpile.com/b/L9hHvg/81T7)[10.1007/s10961-023-10039-5](http://dx.doi.org/10.1007/s10961-023-10039-5)[.](http://paperpile.com/b/L9hHvg/81T7)

[Watters, C. and Lemanski, M.K. (2023) ‘Universal skepticism of ChatGPT: a review of early literature on chat generative pre-trained transformer’, *Frontiers in big data*, 6, p. 1224976.](http://paperpile.com/b/L9hHvg/LxjR)

[Webster-Wood, V.A. *et al.* (2022) ‘Biohybrid robots: recent progress, challenges, and perspectives’, *Bioinspiration & biomimetics*, 18(1). Available at: https://doi.org/](http://paperpile.com/b/L9hHvg/uVfe)[10.1088/1748-3190/ac9c3b](http://dx.doi.org/10.1088/1748-3190/ac9c3b)[.](http://paperpile.com/b/L9hHvg/uVfe)

[Wickberg, A. (2021) ‘Strange natures: Conservation in the era of synthetic biology’, *Global environmental politics*, 21(4), pp. 158–160.](http://paperpile.com/b/L9hHvg/QzNg)

[Wong, F. *et al.* (2023) ‘Discovering small-molecule senolytics with deep neural networks’, *Nature aging*, 3(6), pp. 734–750.](http://paperpile.com/b/L9hHvg/ju8U)

[Wong, F., de la Fuente-Nunez, C. and Collins, J.J. (2023) ‘Leveraging artificial intelligence in the fight against infectious diseases’, *Science*, 381(6654), pp. 164–170.](http://paperpile.com/b/L9hHvg/fxFc)

[Wu, T. *et al.* (2022) ‘Research Trends in the Application of Artificial Intelligence in Oncology: A Bibliometric and Network Visualization Study’, *Frontiers in bioscience* , 27(9), p. 254.](http://paperpile.com/b/L9hHvg/8DaR)

[Xiao, Z. *et al.* (2023) ‘Generative Artificial Intelligence GPT-4 Accelerates Knowledge Mining and Machine Learning for Synthetic Biology’, *ACS synthetic biology*, 12(10), pp. 2973–2982.](http://paperpile.com/b/L9hHvg/WxyO)

[Xu, D. *et al.* (2022) ‘Bibliometric analysis of artificial intelligence for biotechnology and applied microbiology: Exploring research hotspots and frontiers’, *Frontiers in bioengineering and biotechnology*, 10, p. 998298.](http://paperpile.com/b/L9hHvg/3i6a)

[Xu, D. (2023) ‘ChatGPT opens a new door for bioinformatics’, *Quantitative biology (Beijing, China)*, pp. 204–206.](http://paperpile.com/b/L9hHvg/ySMS)

[Yamagata, M. (2023) ‘SynBio: A Journal for Advancing Solutions to Global Challenges’, *SynBio*, 1(3), pp. 190–193.](http://paperpile.com/b/L9hHvg/duUO)

[Yan, X. *et al.* (2023) ‘Applications of synthetic biology in medical and pharmaceutical fields’, *Signal transduction and targeted therapy*, 8(1), p. 199.](http://paperpile.com/b/L9hHvg/58Nf)

[Yeo, H.C. and Selvarajoo, K. (2022) ‘Machine learning alternative to systems biology should not solely depend on data’, *Briefings in bioinformatics*, 23(6). Available at: https://doi.org/](http://paperpile.com/b/L9hHvg/vamg)[10.1093/bib/bbac436](http://dx.doi.org/10.1093/bib/bbac436)[.](http://paperpile.com/b/L9hHvg/vamg)

[Yu, J., Wang, D. and Zheng, M. (2022) ‘Uncertainty quantification: Can we trust artificial intelligence in drug discovery?’, *iScience*, 25(8), p. 104814.](http://paperpile.com/b/L9hHvg/wrAc)

[Zampieri, G. *et al.* (2019) ‘Machine and deep learning meet genome-scale metabolic modeling’, *PLoS computational biology*, 15(7), p. e1007084.](http://paperpile.com/b/L9hHvg/nyJO)

[Zarnack, K. and Eyras, E. (2023) ‘Artificial intelligence and machine learning in RNA biology’, *Briefings in bioinformatics*, 24(6). Available at: https://doi.org/](http://paperpile.com/b/L9hHvg/8aRQ)[10.1093/bib/bbad415](http://dx.doi.org/10.1093/bib/bbad415)[.](http://paperpile.com/b/L9hHvg/8aRQ)

[Zeng, X. *et al.* (2022) ‘Deep generative molecular design reshapes drug discovery’, *Cell reports. Medicine*, 3(12), p. 100794.](http://paperpile.com/b/L9hHvg/z8BD)

[Zeng, X. *et al.* (2022) ‘Regulation and management of the biosecurity for synthetic biology’, *Synthetic and systems biotechnology*, 7(2), pp. 784–790.](http://paperpile.com/b/L9hHvg/0OS1)

[Zeng, Y. *et al.* (2022) ‘Advanced genome-editing technologies enable rapid and large-scale generation of genetic variants for strain engineering and synthetic biology’, *Current opinion in microbiology*, 69, p. 102175.](http://paperpile.com/b/L9hHvg/JbY7)

[Zhang, X.-E. *et al.* (2023) ‘Enabling technology and core theory of synthetic biology’, *Science China. Life sciences*, 66(8), pp. 1742–1785.](http://paperpile.com/b/L9hHvg/Gffq)

[Zhao, H. (2022) ‘Synthetic Biology 2.0: The Dawn of a New Era’, *ACS synthetic biology*, 11(7), p. 2221.](http://paperpile.com/b/L9hHvg/PtLy)

[Zhao, N. *et al.* (2023) ‘Synthetic biology-inspired cell engineering in diagnosis, treatment, and drug development’, *Signal transduction and targeted therapy*, 8(1), p. 112.](http://paperpile.com/b/L9hHvg/OxsG)

[Zhu, X. *et al.* (2023) ‘Current advances of biocontainment strategy in synthetic biology’, *Chinese journal of chemical engineering*, 56, pp. 141–151.](http://paperpile.com/b/L9hHvg/nBMj)
